# cRel and Wnt5a/Frizzled 5 Receptor-Mediated Inflammatory Regulation Reveal Novel Neuroprotectin D1 Targets for Neuroprotection

**DOI:** 10.1007/s10571-022-01231-6

**Published:** 2022-05-27

**Authors:** Jorgelina M. Calandria, Khanh V. Do, Sayantani Kala-Bhattacharjee, Andre Obenaus, Ludmila Belayev, Nicolas G. Bazan

**Affiliations:** 1 Neuroscience Center of Excellence, School of Medicine, Louisiana State University Health New Orleans, New Orleans, LA 70112 USA; 2 Department of Pediatrics, University of California, Irvine, CA 92618 USA; 3 Present Address: Faculty of Medicine, PHENIKAA University, and PHENIKAA Research and Technology Institute (PRATI), A&A Green Phoenix Group JSC, Hanoi, Vietnam

**Keywords:** Non-conventional cytokine, Stroke, Retinal pigment epithelial cells, Ischemia–reperfusion, Neuroprotectin D1, Neuroprotection, Human RPE cells, Inflammatory cytokines, Wnt5a promoter, Uncompensated oxidative stress

## Abstract

**Abstract:**

Wnt5a triggers inflammatory responses and damage via NFkB/p65 in retinal pigment epithelial (RPE) cells undergoing uncompensated oxidative stress (UOS) and in experimental ischemic stroke. We found that Wnt5a-Clathrin-mediated uptake leads to NFkB/p65 activation and that Wnt5a is secreted in an exosome-independent fashion. We uncovered that docosahexaenoic acid (DHA) and its derivative, Neuroprotectin D1 (NPD1), upregulate c-Rel expression that, as a result, blunts Wnt5a abundance by competing with NFkB/p65 on the Wnt5a promoter A. Wnt5a increases in ischemic stroke penumbra and blood, while DHA reduces Wnt5a abundance with concomitant neuroprotection. Peptide inhibitor of Wnt5a binding, Box5, is also neuroprotective. DHA-decreased Wnt5a expression is concurrent with a drop in NFkB-driven inflammatory cytokine expression, revealing mechanisms after stroke, as in RPE cells exposed to UOS. Limiting the Wnt5a activity via Box5 reduces stroke size, suggesting neuroprotection pertinent to onset and progression of retinal degenerations and stroke consequences.

**Graphical Abstract:**

NPD1 disrupts Wnt5a feedback loop at two sites: (1) decreasing FZD5, thus Wnt5a internalization, and (2) by enhancing cREL activity, which competes with p65/NFkB downstream endocytosis. As a result, Wnt5a expression is reduced, and so is its inflammatory signaling in RPE cells and neurons in ischemic stroke.
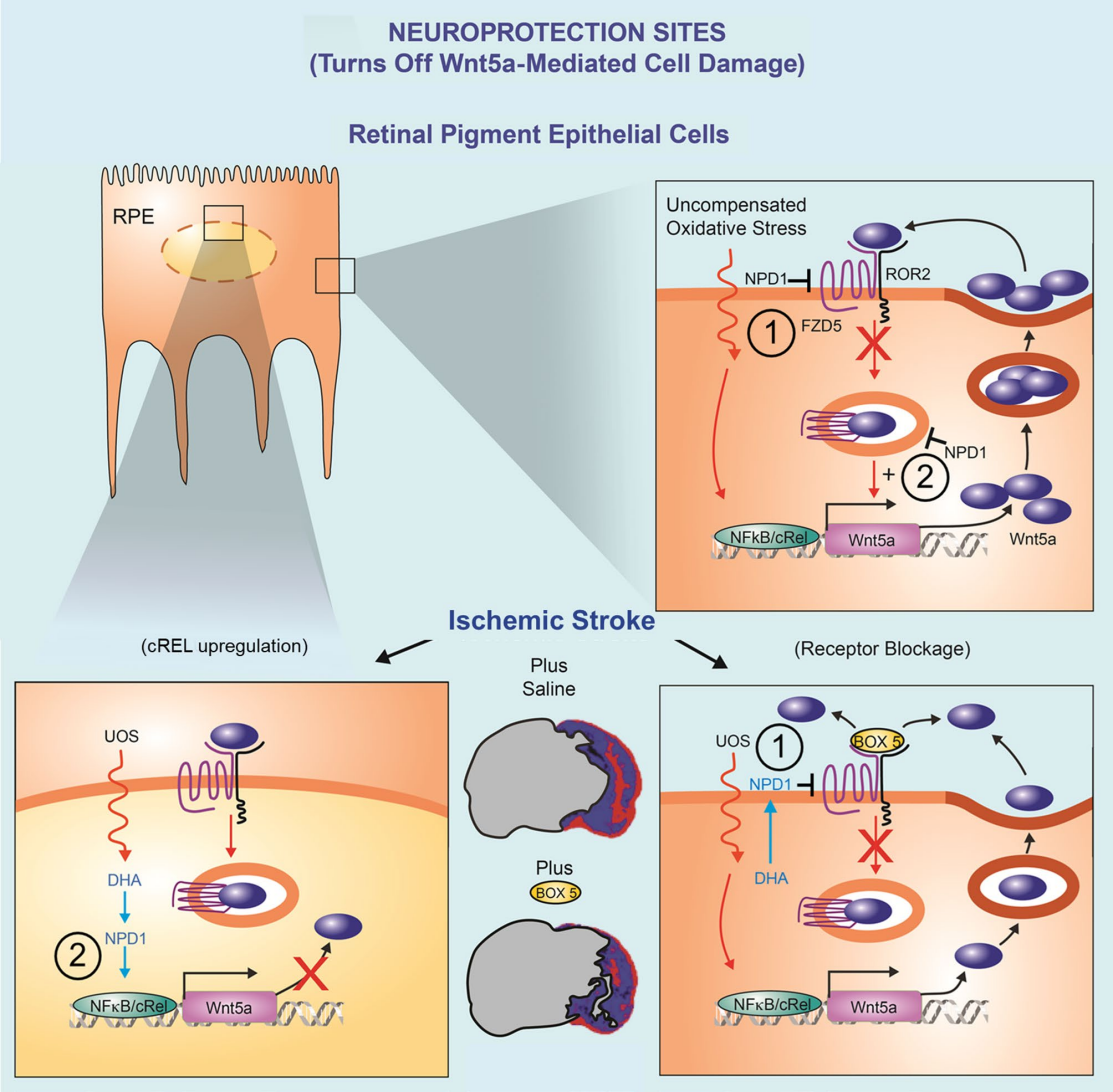

**Supplementary Information:**

The online version contains supplementary material available at 10.1007/s10571-022-01231-6.

## Introduction

Wnt signaling pathways are associated with cellular functions and pathology, including development and cancer (Nusse and Clevers 2017). From the wingless family of ligands, Wnt5a is a secretory glycoprotein that, in certain cases, activates calcium-dependent events via interaction with Frizzled proteins, Ror1/2, RYK, and RTK (De 2011). However, Wnt5a activity is driven by cellular context and is also able to activate beta-catenin via interaction with LRP5/6 (Ring et al. 2014). During tissue morphogenesis and differentiation, Wnt5a is involved in Ca+  +dependent synaptogenesis (Varela-Nallar et al. 2010). Wnt5a has also been associated with inflammatory diseases like rheumatoid arthritis (Sen et al. 2001; Rauner et al. 2012) and atherosclerosis (Ackers et al. 2015). Moreover, Wnt5a/FZD_5_/NFkB interaction is essential in macrophage response to sepsis (Pereira et al. 2008) and innate immune response (Naskar et al. 2014). Activation of NFkB via Wnt5 was also observed in human dental pulp cells (Zhao et al. 2014) and melanoma, the latter by the interaction with ROR1 (Barbero et al. 2019). Because NFkB/p65 is a Wnt5a transcriptional activator (Katula et al. 2012), it heightens its own expression. Thus, we asked if the modulators of uncompensated oxidative stress (UOS) and cell survival (Mukherjee et al. 2004; Calandria et al. 2012), DHA/NPD1 regulate Wnt5a expression and its abundance. Using human primary retinal pigment epithelial (hpRPE) cells, we have addressed events relevant to retinal degenerations by triggering NPD1 synthesis from DHA (Bazan 2006, 2007; Calandria et al. 2009) since these cells support photoreceptor integrity. In these cells, we show that cREL mediates Wnt5a transcriptional regulation by NPD1. In brain ischemia–reperfusion, DHA fosters neuronal survival via NPD1 synthesis that, in turn, activates NFkB/cRel (Calandria et al. 2015). We provide evidence that Wnt5a is upregulated in stroke penumbra and augmented in the bloodstream, favoring activation of immune cells and their recruitment into damaged brain areas. In addition, DHA-decreased bloodstream and penumbra Wnt5a abundance, leading to neuroprotection. Altogether, these results indicate inflammatory modulatory signaling mediated by DHA/NPD1 that engages Wnt5a in responses to neural cell injury.

## Results

### Docosanoids Inhibit UOS-triggered Wnt5a and FZD_5_ Transcription with Concomitant Reduction in Apoptosis

**Fig. 1:**
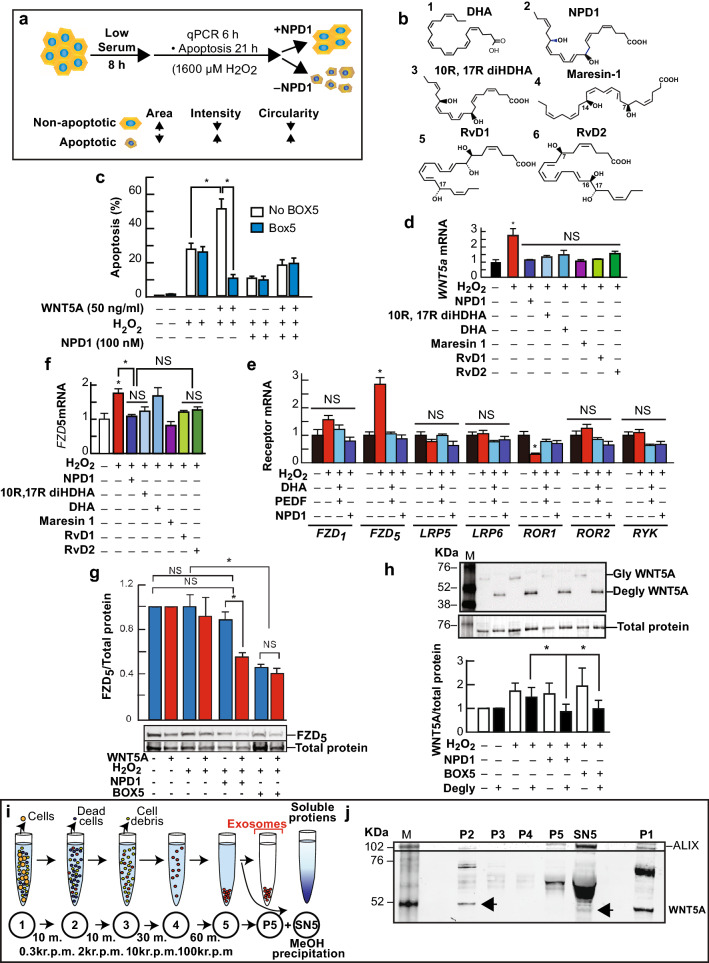
Docosanoids counteract UOS-dependent increase in Wnt5a, FZD_5_ expression, and apoptosis. (**a**) Design to determine apoptotic cells. (**b**) DHA (**1**) and its derivatives: NPD1 (**2**), 10R, 17R diHDHA (**3**), Maresin-1 (**4**), RvD1 (**5**), and RvD2 (**6**) counteracted these effects. (**c, e, and f**) Docosanoids prevented transcription increase in cells undergoing UOS. SYBR green real-time PCR was used to determine semi-quantitatively Wnt5a (**c**) and FZD_5_ € in hRPE cells and receptors linked to Wnt signaling (**f**) in ARPE-19 induce a decrease of UOS-triggered Wnt5a transcription. Standardization was performed using β-actin and GAPDH. (**d**) Wnt5a enhanced cell death triggered by H_2_O_2_. Apoptosis was measured using Hoechst staining and ImageJ using parameters shown in (**a**). (**g**) FZD_5_ was measured in hpRPE cells undergoing UOS treated with 100 nM NPD1 or 100 ng/ml Box5 in the presence or absence of 50 ng/ml Wnt5a. The bands were standardized by total protein stain (see Materials and Methods for description). The bars represent the average of triplicates (Supplementary Fig. S2). (**h**) Deglycosylation of Wnt5a secreted by hpRPE cells. The secreted Wnt5a protein was concentrated from the medium by Chloroform/Methanol precipitation. The pellet was resuspended and digested with N and O-glycosylases (Degly). In parallel, non-digested samples (Gly) were run. Western blots were replicated using two different antibodies and a positive control (Supplementary Fig. S3). (**i-j**) Content of Wnt5a in a medium of human RPE cells in the presence of 1600 µM H_2_O_2_ and 10 ng/ml TNFα. (**i**) Exosome enrichment protocol using ultracentrifugation. (**j**) Content of Wnt5a in the different fractions of the medium. The bars represent the mean of three measurements and the standard error of the mean. *p < 0.05

Retinal pigment epithelia undergoing oxidative stress damage is a key factor in the development of age-related macular degeneration (Do et al. 2019). To model the damage occurring in the retina, hpRPE cells are exposed to H_2_O_2_ and TNFα to induce UOS. Initial observations showed that UOS induced by H_2_O_2_ plus TNFα triggers NPD1 synthesis via 15-lipoxygenase-1 (15-LOX-1) in RPE cells, and the silencing of this enzyme results in NPD1 depletion (Calandria et al. 2009). Six hours after the initiation of UOS, 15-LOX-1 deficient cells displayed a twofold increase in Wnt5a expression (Supplementary Fig. S1a) that was brought down to below control levels by NPD1. Also, DHA plus pigment epithelium-derived factor (PEDF), an agonist of NPD1 synthesis (Mukherjee et al. 2007), prevented Wnt5a upregulation in hpRPE cells (Supplementary Fig. S1b). To test the idea that other docosanoids (Fig. 1b1–6) downregulated Wnt5a transcription, we confronted hpRPE cells for 6 h using 1600 µM H_2_O_2_ and 10 ng/ml TNFα in the presence or absence of DHA (Fig. 1b1), NPD1 (Fig. 1b2), 10R, 17R diHDHA (Fig. 1b3), Maresin-1 (Fig. 1b4), RvD1 (Fig. 1b5) or RvD2 (Fig. 1b4). All docosanoids decreased the expression of Wnt5a to control levels (Fig. 1c). Recombinant Wnt5a potentiated cell death by UOS in ARPE-19 cells (Supplementary Fig. S1c) and in hpRPE (Fig. 1d) measured as depicted in Fig. 1a. A hexapeptide that corresponds to the amino acid portion 332 to 337 of Wnt5a with the t-Boc substitution in the N-terminal (t-Boc-NH-Met-Asp-Gly-Cys-Glu-Leu-CO_2_H), Box5 is a Wnt ligand analog that blocks binding to receptors (Jenei et al. 2009). Box5 or NPD1 hindered apoptosis by UOS in the presence of the Wnt5a. Wnt5a alone had no effect on RPE resting cells (Supplementary Fig. S1c), implying that the Wnt5a acts through a different pathway in cells undergoing UOS.

To assess the co-regulation of the transcription of Wnt5a receptors and co-receptors (FZD_1_ and _5_, LRP5/6, RYK, and ROR1/2) (Mikels and Nusse 2006), we assayed their expression by SYBR green-based real-time PCR. hpRPE were exposed to UOS; FZD_5_ mRNA rose twofold and then dropped to control levels when docosanoids were added (Fig. 1e). DHA leads to the synthesis of 10R, 17R diHDHA, Maresin 1, RVD1, RVD2, and NPD1 (Fig. 1b1–6); however, this fatty acid alone did not affect FZD_5_ expression, implying that its conversion to lipid mediators is required to counteract the effect of UOS on FZD_5_. Unlike FZD_5_, other Wnt signaling receptors: FZD_1_, LRP5, LRP6, ROR2, and RYK, remained unchanged, although ROR1 expression was reduced under UOS and re-established by NPD1 (Fig. 1f). To determine the FZD_5_ protein availability, a western blot analysis was performed in cell extracts from hpRPE cells exposed to UOS 1 + / − NPD1 or Box5 when Wnt5a was added. Contrary to mRNA levels (Fig. 1e, f), cells exposed to UOS displayed a similar FZD_5_ content to controls, pointing out that receptor regulation by degradation (Fig. 1g, Supplementary Fig. S2). The presence of Box5 decreased by half FZD_5_ abundance, even in the presence of Wnt5a, showing that the binding inhibition of the true ligand enhances receptor degradation as well. Intriguingly, when NPD1 was added in the absence of Wnt5a, no differences in the FZD_5_ receptor were observed, although, when the ligand was present, a steep decrease in the receptor content was evident (Fig. 1g, Supplementary Fig. S2). This likely signifies that NPD1 enhances FZD_5_ degradation via internalization of the FZD_5_/Wnt5a complex.

Wnt5a displayed in western blots, besides the light band that ran between 52 and 38 KDa, a higher molecular mass band with all the antibodies used in this study. Since Wnt5a is glycosylated and palmitoylated (Kurayoshi et al. 2007), we tested if the band above 52KDa was a highly glycosylated form of the ligand by total deglycosylation (Degly) of the precipitated medium in comparison to non-deglycosylated samples (Gly). Deglycosylation followed the pattern of intensity observed when the cells were exposed to UOS + / − NPD1 or Box5 (Fig. 1h). However, the samples that were not digested showed a different pattern, likely due to the different affinity of the antibodies to the modified Wnt5a. Overexpression of Wnt5a showed that Wnt5a increases after deglycosylation, but no noticeable change in the hyperglycosylated state by the antibody takes place (Supplementary Fig. S3). This may imply that hyperglycosylated secreted protein may be a mechanism by which the cell modulates ligand activity.

It has been proposed that active Wnt5a was released in exosomes (Gross et al. 2012). To test whether Wnt5a was released by hpRPE cells in exosomes, we analyzed medium (after ultracentrifugation) from cells undergoing UOS (Fig. 1i). Western blot analysis of the first pellet after spinning at 300 rpm (dead cells pull-down) showed a 35 KDa band as that of mature Wnt5a. The band's pattern closely resembles the one in whole cells (Fig. 1j). Pellets of 2000 and 10,000 rpm containing cell debris (Fig. 1i) displayed no bands, and the pellet from ultracentrifugation at 100,000 rpm (exosomes) lacked a 42 KDa band. The supernatant was then precipitated using Methanol/Chloroform, and a 42 KDa emerged, indicating that most of Wnt5a is not contained in exosomes. However, a band above 52KDa appeared in the pellet obtained in the 100 K x g centrifugation and a very intense band in the supernatant that was further precipitated, indicating that the glycosylated forms were present in exosomes but mainly in soluble form. These observations demonstrate that Wnt5a is mainly released in an exosome-free manner by hpRPE cells undergoing UOS.

These results demonstrate that NPD1 downregulates the transcription of Wnt5a and FZD_5_ and decreases the availability of FZD_5_ receptor protein when Wnt5a is added to prevent the ligand-receptor binding. In addition, Wnt5a extracellular availability is mainly endosome-free and is controlled by NPD1.

### Wnt5a Colocalizes with FZD_5_ and is Internalized via Clathrin

**Fig. 2:**
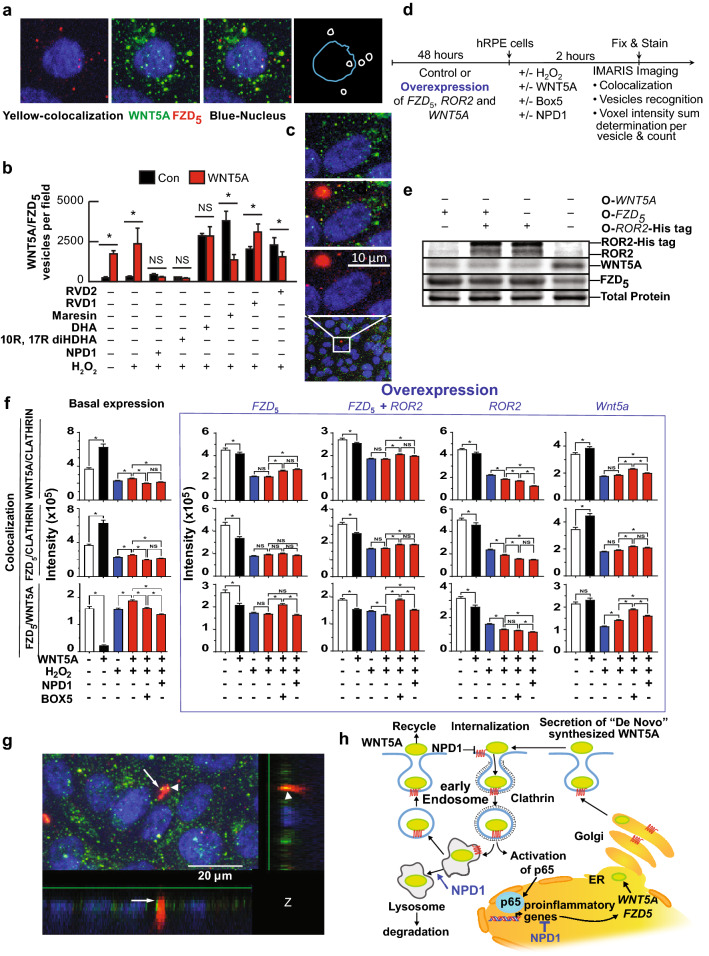
Wnt5a and FZD_5_ are internalized via CME. (**a**) Blow-up of a single cell showing vesicles positives to Wnt5a (green), FZD_5_ (red), or both (yellow). The fourth panel shows a nucleus drawing (blue) and the position of vesicles in which FZD_5_ colocalizes with Wnt5a. (**b**) Quantification of colocalized spots in RPE cells undergoing UOS ± lipids in Fig. 1b. Pearson colocalization coefficient was plotted in fig. S8D. Bars: mean of 3 measurements and standard error of the mean. * p < 0.05. (**c**) Blow-up of a cell showing large cluster of Wnt5a signal (red) present most frequently in certain treatments (Supplementary Fig. S8c). (**d**) Timeline of experiment depicted in **e** and **f**. (**e**) Western blot analysis of ROR2, FZD_5_, and Wnt5a in hpRPE cells overexpressing ROR2-His tag ORF, FZD_5_ ORF, ROR2-His tag and FZD_5_ ORFs together and Wnt5a. Duplicates of whole membranes are shown in Supplementary Fig. S4. (**f**) IMARIS analysis of the vesicles observed by immunocytochemistry targeting Wnt5a, FZD_5_, and Clathrin. The sum of the intensity for the colocalization of the signal observed for Wnt5a/Clathrin, FZD_5_/Clathrin, and FZD_5_/Wnt5a was measured with the Spots module using batch processing. Each spot corresponds to one individual or clusters of vesicles observed in hpRPE cells under UOS in the presence or absence of NPD1or Box5 and Wnt5a in the cells expressing the ORFs (open reading frame) tested by WB in (**d**). Five fields per well were averaged and computed to obtain the bars plotted. Two ways ANOVA and Tukey's HSD was applied to determine the pairwise comparison significance. The number of Spots plots for each histogram is depicted in Supplementary Fig. S5. (**g**) Vesicle-like signal in the Z axes of the Z-stack. Whole arrow shows a fusion between an FZD_5_ and Wnt5a positive to a large Wnt5a-positive cluster. Arrowhead shows an already fused colocalized cluster. (**h**) Model of internalization and recycling of Wnt5a and FZD_5_ to activate NFkB/p65. The bars represent the mean of three measurements and the standard error of the mean. *p < 0.05

To assess colocalization of Wnt5a and FZD_5_, hpRPE undergoing UOS + / − Wnt5a were immunostained for FZD_5_ and Wnt5a. Confocal z-stacked images were analyzed using IMARIS 9.3 software to determine the distribution of the colocalized signal. Figure 2a upper panels depict the signals of FZD_5_ (red) and Wnt5a (green), the colocalization of both (yellow), and in the last panel, the drawing shows only the nucleus and the punctuated signal with colocalization. The punctuated nature of the signal resembled vesicular internalization (Feng and Gao 2015). The analysis using IMARIS software was then used to count vesicles that contained colocalized signals of Wnt5a and FZD_5_ in hpRPE undergoing UOS with Wnt5a ± NPD1 or other lipid mediators for 2 h. The mean of vesicles/field that showed colocalization of FZD_5_/Wnt5a was increased with the addition of the ligand in the control and UOS cells (Fig. 1b), indicating that the Wnt ligand promotes its own internalization. NPD1 and its bioactive stereoisomer 10R, 17R diHDHA decreased the number of vesicles per field in the presence or absence of Wnt5a but not other docosanoids and related bioactive lipids tested (Fig. 1b).

We hypothesized that Wnt5a/FZD5 was internalized via clathrin-mediated endocytosis as other members of the Wnt pathway (Albrecht et al. 2021); thus, the vesicles load was measured using the IMARIS function sum of the intensity for the pair colocalization of Wnt5a-FZD_5_, FDZ_5_-Clathrin and Wnt5a-Clathrin. In parallel, hpRPE cells overexpressing FZD_5_, FZD_5_ + ROR2, ROR2, or Wnt5a were analyzed (Fig. 2d, e; Supplementary Fig. S4). ROR2 was included as a co-receptor of FZD_5_ because it was proposed as a proinflammatory receptor for Wnt5a in atherosclerosis and lung COPD (Ackers et al. 2020; Zou et al. 2020). Wnt5a overexpression showed a high number of vesicles that colocalize with FZD_5_/Wnt5a signal as well as with Wnt5a- and FZD_5_-positive vesicles even in the presence of NPD1 (Supplementary Fig. S5). Interestingly, FZD_5_ overexpression displayed a low number of FZD_5_ vesicles while the overexpression of ROR-2 induced a steep increase in the Wnt5a/FZD_5_- and Wnt5a-positive vesicles in controls and NPD1-treated cells, but more so in cells undergoing UOS (Supplementary Fig. S5). The colocalization intensities for FZD_5_/clathrin, Wnt5a/clathrin, and Wnt5a/FZD_5_ vesicles in cells overexpressing FZD_5_, ROR2 and both together + / − Wnt5a showed the same pattern where the addition of Wnt ligand decreased the load of the vesicles, whereas the control (receptors baseline expression) and the overexpression of the Wnt5a depicted the opposite trend (Fig. 2f). NPD1 decreased the colocalization of FZD5 and Wnt5a in hpRPE cells undergoing UOS in the presence of Wnt5a (Fig. 2f, first column, lower histogram). When FZD5 was overexpressed alone or with ROR2, that effect was abolished, even bringing the levels of intensity when NPD1 was present above the UOS plus Wnt5a (Fig. 2f, second and third column, lower histograms). The same effect was observed when Wnt5a was overexpressed (Fig. 2f, fifth column, lower histogram). The intensities of the colocalization of the three pairs and the comparisons between UOS Wnt5a and UOS Wnt5a plus NPD1 remained invariable in ROR2 overexpressing hpRPE cells (Fig. 2f, fourth column); thus, NPD1 is not operating on this protein to modulate Wnt5a signaling. Clathrin coated are early endocytosis vesicles, and their distribution displayed remarkable differences between cells resting and undergoing UOS, confirming the observations made in Fig. 1d that show Wnt5a pathways differed in the presence of stress signaling.

It was proposed that Wnt5a/FZD_5_ internalization proceeds via Clathrin-coated vesicles in the formation of a signalosome when activation of β-catenin occurs. Because the signaling observed in the absence of stress differs greatly from the one in the presence of UOS, we tested whether Wnt5a by itself activates β-catenin using TOP Flash/FOP flash reporter system in hpRPE cells undergoing UOS ± NPD1 (Supplementary Fig. S6). In the absence of Wnt5a or Wnt3a, β-catenin activity was not significantly altered by UOS or NPD1 (Supplementary Fig. S7). When Wnt3a was added, luciferase rose almost twice, which is consistent with the β-catenin co-activation of TCF/LEF reporter system. Wnt5a alone did not affect β-catenin (Supplementary Fig. S7), so in this case, Wnt5a signaling in the absence of activated β-catenin does not involve TCF/LEF-related gene expression.

The vesicles observed in the immunocytochemistry showed different size vesicles (Fig. 2g, Supplementary Fig. S8) compatible with the model presented in Fig. 2h and proposed elsewhere (Brunt and Scholpp 2018), where clathrin-dependent endocytosis (CEM) is responsible for the internalization of Wnt5a/FZD5. The heterogeneity of the signal within the vesicles, the size, and the intensity observed by immunocytochemistry (Supplementary Fig. S8) lead us to propose that there is a sorting system in which the large vesicles that are labeled positive for Wnt5a (Fig. 2g) are probably targeted for degradation via lysosome (Fig. 2h). NPD1 decreases the internalization of Wnt5a, reducing the availability of its receptor, FZD_5_.

### Wnt5a-Dependent Activation of NFkB Requires FZD_5_ and ROR2

**Fig. 3:**
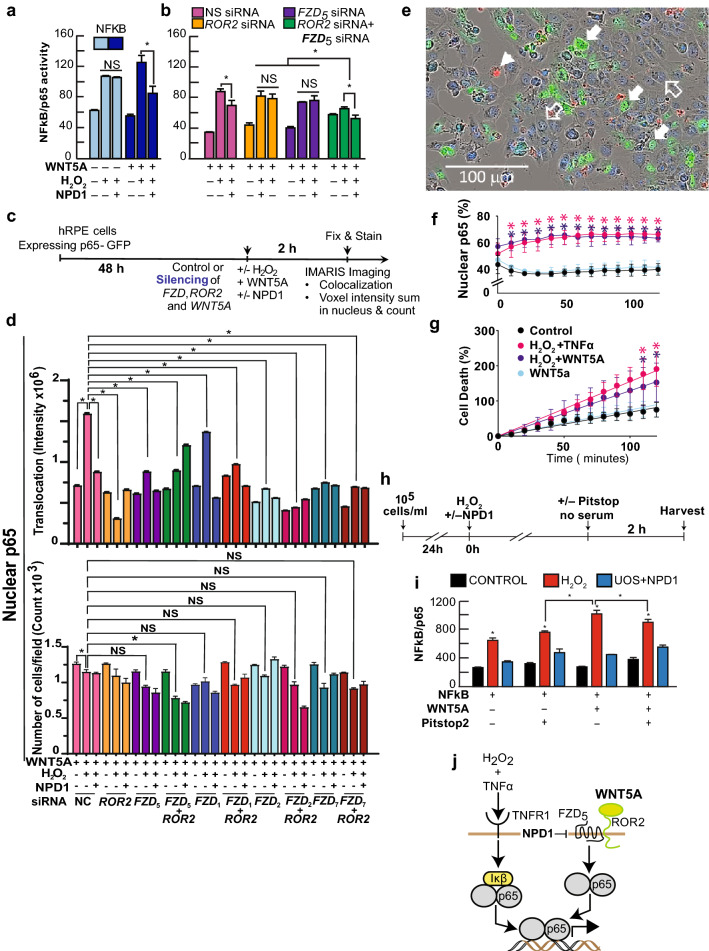
Wnt5a triggers the activation of p65 in human RPE cells undergoing UOS. (**a, b**) Luciferase assay using NFkB/p65 binding site construct (Supplementary Fig. S6) to determine its activation in the presence or absence of Wnt5a in hpRPE cells undergoing UOS plus/minus NPD1. The standardization was made co-expressing constitutively GFP. (**b**) hRPE cells were co-transfected with the NFkB/luciferase plasmid and siRNA targeting FZD_5_ and ROR2 separately and together or control non-specific siRNA. UOS was induced ± 100 nM NPD1 and Wnt5a. The yield of expression for FZD_5_ and ROR2 are presented in Supplementary Fig. S9. Bars show the mean of three measurements and the standard error of the mean. *p < 0.05. (**c, d**) Specificity of Wnt5a/FZD receptor activity on the activation of p65. (**c**) Timeline showing experimental design. (**d**) Silencing of FZD_1_, FZD_2_, FZD_5_, and FZD_7_ alone or together with ROR2 (Supplementary Fig. S10) in hpRPE cells expressing p65-GFP were treated with 50 ng/ml Wnt5a, in the presence or absence of H_2_O_2_ to induce uncompensated oxidative stress and NPD1 for two hours. Nuclear p65 was assessed by analyzing confocal Z-stack images with Imaris 9.8 to determine nuclear translocation (colocalization of GFP and Hoechst staining), Intensity (upper histogram), and number of cells (lower histogram). (**e–g**) In vivo monitoring of p65 nuclear translocation in resting hRPE cells or undergoing UOS cells and exposed to Wnt5a. hRPE cells expressing human p65fused to GFP incubated for 120 min in an Incucyte SX5 Life-cells Analysis instrument and registered every 10 min. TNFα was used as a positive control. (**e**) Cell death using Propidium Iodide (PI) (orange), total cell staining DRAQ5 (blue), and p65-GFP (green). Solid white arrows = Nuclear GFP (computed as DRAQ5/p65-GFP overlapped signal); solid white arrowheads = PI dying cells, open white arrows = normal cells. (**f**) Upper panel shows the percentage of cells depicting nuclear p65, (**g**) lower panel shows the percentage of dead cells that became permeable to PI staining. Nine fields per well for a total of 4 wells per condition were computed (**f, g**). (**d, f, and g**) 2-way ANOVA and Tukey HSD multiple comparisons were applied to obtained pairwise significance. *p < 0.05. (**h, i**) Pitstop2 halts activation of NFkB. (**h**) Experimental design. (**i**) Luciferase Reporter assay of three NFkB/p65 binding sites in tandem driving the expression of luciferase ORF; the construct was depicted in Supplementary Fig. S6. NPD1 = 100 nM and 1600 µM H_2_O_2_. (**j**) Model of alternative pathways of activation of p65 and the action of NPD1

Wnt5a was proposed to interact with FZD_5_ and ROR2 to trigger inflammatory gene expression via NFkB activation (Naskar et al. 2014; Sato et al. 2015). To determine the contribution of FZD_5_ and ROR2 in the NFkB activation, we silenced the receptor and co-receptor separately and together (Supplementary Fig. S9) in human RPE cells transfected with a construct that encompassed three p65 high affinity binding sites in tandem, driving the expression of the luciferase reporter gene (Supplementary Fig. S5). hpRPE cells exposed to UOS showed increased luciferase activity, and NPD1 did not affect NFkB/p65 as shown previously (Fig. 3a) (Calandria et al. 2015). Exposure to 2 h of Wnt5a heightened NFkB activation in the presence of UOS, and NPD1 reduced luciferase activity (Fig. 3a). These results suggest that the activation of NFkB/p65 via Wnt5a is exerted by a different signaling pathway than the one triggered by UOS/TNFα, and in this case, the effect is responsive to NPD1 (Fig. 3a). hpRPE cells transfected with control siRNA showed the same pattern of NFkB activity seen in Fig. 3b. ROR2 or FZD_5_ siRNAs separately abolished the difference between UOS and UOS + NPD1 (Fig. 3b), but the co-transfection of both siRNAs against ROR2 and FZD_5_ together induced a deeper decrease in p65/NFkB activation that was not affected by NPD1 effect, on the contrary, it potentiated its activity (Fig. 3b). When Wnt5a was added to the double knockdown cells, the NFkB activity did not differ from controls, but it did show a slight yet significant difference with the UOS-treated cells, showing that both receptor and co-receptor are required to activate NFkB/p65.

To determine specificity of FDZ_5_ and ROR2 in NFkB activation by Wnt5a, cells were silenced by other FZD receptor candidates (FZD_1_, FZD_2_, and FZD_7_) separately and along ROR2 (Supplementary Fig. S10). Another two candidates, FZD_4_ and FZD_8_ (Dijksterhuis et al. 2015), are not expressed in human RPE mature cells. The discovery came to us after silencing both receptors because the intensity and count of p65 rendered no difference from the control (Supplementary Fig. S11). In addition, quantification of FZD_4_ and FZD_8_ by the real-time PCR yielded no amplification, and an RNAseq database from three types of RPE (ARPE-19 cell line, hpRPE from 19-year-old male used in this manuscript, and hpRPE from male 49-year-old cells) showed FDR cero. The hpRPE cells knocked down for all the receptors and combinations and expressing p65 fused to GFP were exposed to UOS plus Wnt5a in the presence or absence of NPD1 for 2 h (Fig. 3c, d). Z-stacks obtained by laser confocal microscopy were processed using Imaris 9.8 to determine the intensity of GFP in the nucleus and the number of nuclei per field that carry the signal (Fig. 3d); thus, NFkB activation was measured as both p65 nuclear translocation and number of cells that responded to the treatment (Fig. 3d). The strongest combined decrease in nuclear p65 (count and intensity) only was achieved when FZD_5_ and ROR2 were silenced together (Fig. 3d). FZD_2_ and FZD_7_ knockdown depicted a sharp reduction of p65 in nuclei, and for this reason, we cannot rule out a contribution from FZD_2_ and FZD_7_; however, as the number of positive nuclei was not significantly different from the negative control silence in both cases, it is less likely that they are leading the main modulation (Fig. 3d).

In addition, hpRPE cells were transfected with p65 fused to GFP and monitored in real-time using Incucyte microscopy after the addition of H_2_O_2_ plus TNFα as a positive control, H_2_O_2_ plus Wnt5a, Wnt5a alone, and a vehicle control to set the baseline (Fig. 3e, f). In parallel, the apoptosis was tracked using Propidium Iodide, which penetrates the cells only when cell death occurs (Fig. 3g). Initially, 50% of the cells contained p65 in their nuclei, but after 10 min of monitoring, the cells treated with H_2_O_2_ plus TNFα and plus Wnt5a stabilized at the same level, and the cells that only received Wnt5a reached control levels (Fig. 3f). Cell death was also segregated into the same two different groups, raising linearly throughout the two hours (Fig. 3g). H_2_O_2_ plus TNFα and H_2_O_2_ plus Wnt5a-treated cells differed from their controls, showing a trend of separation and a significant difference at 110 and 120 min. These results demonstrate that wnt5a alone does not induce damage on hpRPE cells and does not activate NFkB in the absence of stress and confirm previous observations (Supplementary Fig. S1c).

Altogether, the results point out that Wnt5a activates NFkB/p65 in an FZD_5_/ROR2-dependent manner and NPD1 indirectly decreases the NFkB/p65 activity. We propose a model in Fig. 3j highlighting the differences between activation of NFkB/p65 by TNFα (NPD1 insensitive) and Wnt5a (NPD1 sensitive).

### Wnt5a/FZD_5_ Clathrin-mediated Endocytosis is Required for NFkB Activation

Wnt5a signaling required FZD_5_ and ROR2 to activate p65/NFkB under UOS conditions (Fig. 3a-g). To determine whether clathrin-mediated endocytosis was required for the activation of p65/NFkB via Wnt5a, hpRPE cells were transfected with the NFkB/p65 reporter construct with UOS ± 200 nM NPD1 plus 50 ng/ml Wnt5a (Fig. 3h). The addition of Pitstop2 that competitively inhibits clathrin terminal domain in cells undergoing UOS in the presence of Wnt5a decreases the activation of p65/NFkB, suggesting that the internalized Wnt5a is responsible for NFkB activation (Fig. 3h).

### NPD1 Stimulates cRel Binding to Wnt5a Promoter A That in Turn Prevents Transcriptional Activation

The observation that NPD1 reduces the activity of NFkB/p65 in cells undergoing UOS in the presence of Wnt5a when FZD_5_ and ROR2 are knocked down points to the lipid mediator operating via an additional effector other than p65. In Calandria et al. 2015, we proposed that cREL was activated by NPD1 and induced a displacement of p65 from the same binding site to modulate the transcription of BIRC3. Here we used the Wnt5a promoter to test the hypothesis that NPD1 is modulating the binding of p65 via cREL.

**Fig. 4:**
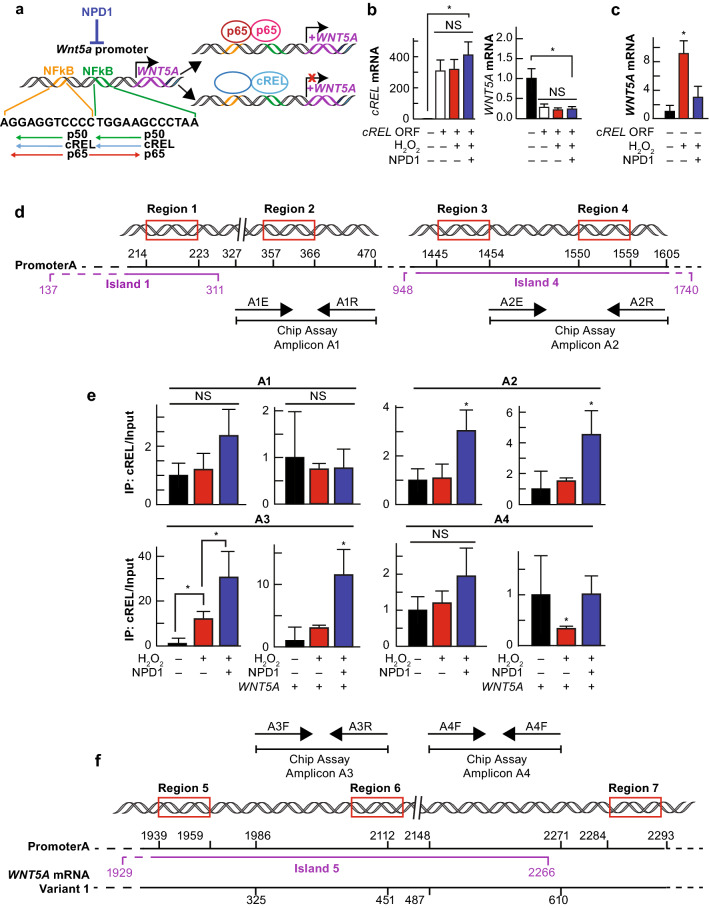
NPD1-dependent cRel binding to promoter A decreases Wnt5a expression. (**a**) Hypothesis of the role of cREL in the modulation of the expression of Wnt5a. (**b, c**) Quantification of cRel (**b**) and Wnt5a (**C**) mRNA by the means of SYBR green-based real-time PCR in hpRPE cells undergoing UOS, ± NPD1. (**B**) Cells transfected with cREL ORF were exposed to UOS in the presence or absence of NPD1 for 4 h. cREL (left) and Wnt5a (right) mRNA was quantified by real-time PCR. (**c**) Wnt5a mRNA quantification of non-transfected cells (Control for **b**). (**d, f**) Possible regulation of NFkB sites by cRel: in silica analysis of Wnt5a promoter (Katula et al. 2012) showing that the two binding sites for NFkB have high affinity for p65, p50, and cRel. The cartoon shows the possible direction in which transcription factors act. NFkB site prediction is in Supplementary Table S1. Other possible NFkB binding sites were detected by TRED. Region 2 corresponds to upstream NFkB binding site, and Region 6 to the downstream binding site depicted in** d** and **f**. Regions 1, 3, 4, 5, and 7 showed up in the general TRED search with high score (Supplementary Table S1) for the three NFkB. Four amplicons were designed close to or sitting on these regions to assess each site. CpG islands that encompass the putative binding sites are depicted in purple (Supplementary Table S2). (**e**) SYBR green-based real-time PCR using as template the proteinase digested genomic DNA fragments resulting from micrococcal DNAs digestion and cRel pull-down. 1600 µM H_2_O_2_ plus 10 ng/ml TNFα. NPD1 = 100 nM and Wnt5a 50 ng/ml unless stated otherwise. The bars represent mean of three measurements and standard error of the mean. *p < 0.05

The Wnt5a gene is under the regulation of 2 promoters located in exon1a and exon2 (Vaidya et al. 2016). Promoter A drives the expression of the largest form, variant-1, and contains at least two binding sites for NFkB (Katula et al. 2012). In-silico analysis using TRED software showed that both sites have a high affinity for 3 NFkB members: p50, cRel, and p65. Downstream, p50 and cRel binding are opposed to the p65 site, signifying that p65 and cRel activity compete to oppose each other (Fig. 4a and Supplementary Table S1). To confirm a direct link between cRel expression and Wnt5a transcription, we over-expressed the transcription factor in hpRPE (Fig. 4b). cRel overexpression decreased Wnt5a mRNA (Fig. 4b). The increase in cRel availability dominantly shut off Wnt5a expression regardless of the treatment. Altogether, the data indicate that cRel blocks Wnt5a expression triggered by UOS, which may be a key modulatory NPD1 function.

Based on the regions found to bind NFkB members (Supplementary Table S1), we designed four sets of primers that bound in the proximity or within the regions of interest to perform SYBR green-based real-time PCR detection (Fig. 4d, f and Supplementary Table S3). Figure 4d, f and Supplementary Table S2 also show regions of high probability for methylation, a mechanism proposed to block Wnt5a transcription (Vaidya et al. 2016). Micrococcal DNase digested genomic DNA fragments from UOS or UOS-plus-NPD1-treated RPE cells, ± rWnt5a, were pulled down by a cRel antibody and used to test the four sets of primers. Amplicon A1, which is localized close to Region 1 and overlaps Region 2 cRel/p65 binding sites (Fig. 4d), showed no differences between treatments; thus, no differential binding of cRel to the genomic DNA in the presence of Wnt5a and NPD1 (Fig. 4e). Methylation of Region 1 may occur since it was predicted by the Methprimer software (Supplementary Table S5). The amplicon 2 encompassing Regions 3 and 4 displayed twice the amount of cRel bound to genomic DNA under NPD1 treatment. Within the proximity of Region 5 and overlapping Region 6 (Fig. 4f), the amplicon 3 displayed the largest differences between treatments, reaching more than 20-fold when NPD1 was added to RPE cells undergoing UOS in the absence of Wnt5a and tenfold when Wnt5a was present. The results obtained for amplicon 3 shows evidence of competition between the Wnt5a-activated p65 and NPD1-activated cRel (Fig. 4e). Finally, Amplicon 4, which is located upstream of Region 7, showed no differences in the absence of Wnt5a but did show a decrease in RPE cells undergoing UOS in the presence of rWnt5a. NPD1 restored the cRel binding to control levels; hence, in the presence of NPD1, cRel displaces the initially bound p65 (Fig. 4e, f). These data indicate a binding interactive competition between p65 and cRel that depends on availability and other factors, such as methylation for NPD1-mediated cell survival.

### Ischemic Stroke Activates Wnt5a Expression: DHA and BOX5 elicit Neuroprotection

RPE cells are embryonically originated from the neuroectoderm through successive neural developmental steps and terminally differentiated into epithelial-like type. As such, they retain certain neural characteristics (Bazan 2007). Taking advantage of this distinctive feature, we decided to test the Wnt5a paradigm studied in RPE cells in a model portraying oxidative stress damage, like ischemia–reperfusion (I-R) (Belayev et al. 2017).

**Fig. 5:**
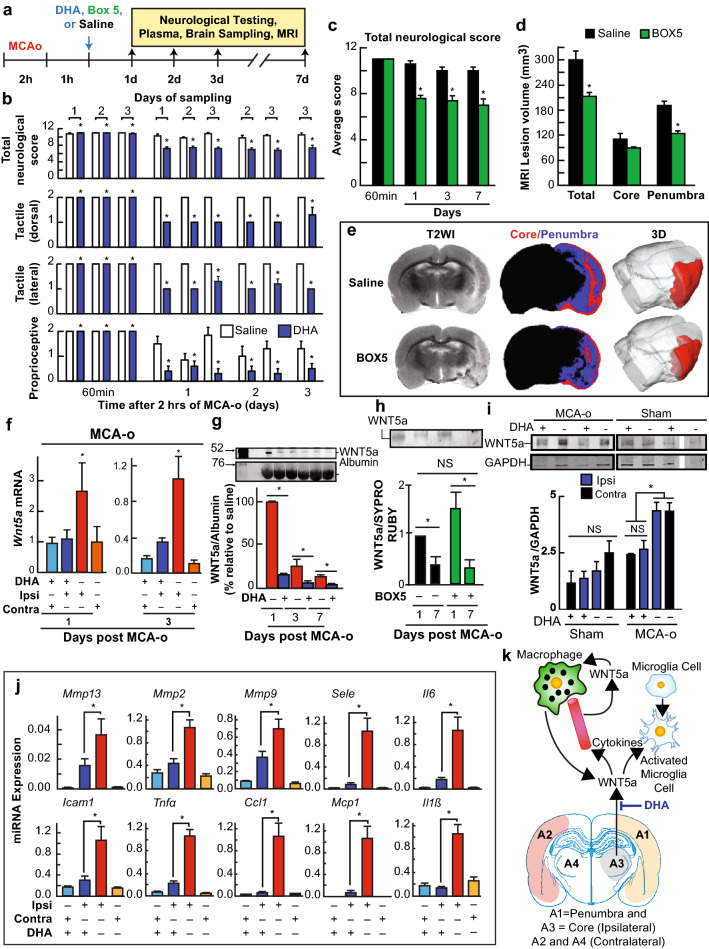
Neuroprotection by DHA prevents Wnt5a overexpression and secretion in response to brain ischemia–reperfusion and by Box5 that blocks its action. (**a**) DHA, Box5, or saline were administered at 1 h after 2 h of MCAo, and rats were sacrificed on days 1, 2, 3, or 7. (**b**) Neurological recovery. Total score (normal score = 0, maximal deficit = 12), tactile placing (dorsal, lateral, proprioceptive reactions; normal score = 0, maximal deficit = 2). DHA or saline was administered at 1 h after 2 h of MCAo, and rats were sampled on days 1, 2, 3, or 7. Values are mean ± SD; n = 4 rats/ group. *Significantly different from saline group (p < 0.05, repeated measures ANOVA followed by Bonferroni tests). (**c-e**) 400 µg Box5 IV administration, 1 h after MCAo (**c**) Total neurological score at days 1, 3, and 7; (**d**) MRI quantification at day 7 of lesion volume depicting total, core and penumbra and (**e**) representative coronal sections showing T2-weighted image (T2WI), the defined core and penumbra region (red and blue, respectively) in the second column; and a 3D reconstruction of the lesion. (**f**) Wnt5a mRNA assessment by SYBR-green real-time PCR in rat cortex Ipsilateral (Ipsi) or contralateral (Contra) of MCAo, treated with saline (vehicle) or DHA. (**g, h**) Wnt5a protein in plasma 2 h after MCAo with DHA (N = 4); (**g**) or Box5 (N = 4); (**h**) at 1, 3 and 7 days post-surgery. (**i**) Representative bands are depicted above the plot. (**j**) Wnt5a and NFkB linked gene expression in MCAo. MCAo and saline (vehicle) and DHA for 3 days (N = 3); each reaction was run in triplicate. mRNA measured using SYBR green RT-PCR. Color-coded region to test gene expression is depicted. Bars represent t mean of 3 measurements and standard error of the mean. *p < 0.05. (**k**) Wnt5a inflammatory signaling after increased abundance due to ischemia/reperfusion

Intravenously (IV), DHA reduces I-R brain damage. To test whether IR increases Wnt5a secretion and signaling, we induced stroke in rats by middle cerebral artery occlusion (MCAo) for 2 h and, 1 h later, injected IV saline (vehicle) or DHA. Neurological scores, tactile and proprioceptive tests 1, 2, 3, and 7 days after MCAo showed severe neurological impairments in saline-treated rats (Fig. 5a, b). DHA treatment improved neurologic scores, including tactile (dorsal and lateral) and proprioceptive forelimb placing reactions (Fig. 5b). To test whether Wnt5a is involved in post-ischemia–reperfusion damage, we injected the receptor blocker Box5 after MCAo (Fig. 5a) and found neurological protection remarkable similar to that obtained by DHA treatment (Fig. 5d). Moreover, MRI illustrated decreased volume of brain damage (Fig. 5d, e) resembling those observed by DHA injection (Belayev et al. 2017). Furthermore, we assessed Wnt5a mRNA in A1 penumbra, A3 stroke core, and as control A2 and A4 that correspond to contralateral parts of A1 and A3 (Fig. 5k). The penumbra, an area surrounding the ischemic core, is subject to moderate damage and is more likely to survive ischemic-reperfusion when treatment is applied. We found that the Wnt5a mRNA was increased in the ipsilateral hemisphere 1–3 days post-surgery and that DHA blocked this surge (Fig. 5f). After stroke, both hemispheres work synergistically to overcome damage (Buga et al. 2008). In this case, the increase in Wnt5a mRNA level was detected only in the ipsilateral hemisphere; the contralateral showed no surge in Wnt5a expression 1–3 days after surgery, denoting a local induction of mRNA expression. However, Wnt5a protein abundance showed that the levels of ipsilateral and contralateral hemispheres A1 and A2 did not differ from one another, and they were both high in saline and low in DHA-treated animals (Fig. 5i and Supplementary Fig. S14). In addition, Wnt5a was enhanced in blood after MCAo 1-day post-stroke and was decreased at day 3 (Fig. 5g and Supplementary Fig. S12). Impairment of the interaction Wnt5a/receptor with Box5 that induced changes in the size of the infarct observed in the MRI (Fig. 5d, e) and an improvement in neurological score (Fig. 5c) failed to reduce the plasma Wnt5a content, suggesting a difference in the action between DHA and Box5. In agreement with the activation of NFkB/p65, the expression of NFkB-activated inflammation mediators IL6, TNFα, CCL1, MCP1, and IL1β follows the same trend as Wnt5a after DHA (Fig. 5j and Supplementary Fig. S13). MMP13, MMP2 and MMP9 expression is enhanced when Wnt5a-ROR2 is activated (Yamagata et al. 2012). The three mRNAs showed the same expression trend as Wnt5a (Fig. 5j), hence the activation of ROR2 by Wnt5a. Other genes involved in inflammatory signaling that are known to be activated by Wnt5a (Kim et al. 2010), such as E-selectin and ICAM-1, were found to follow the same pattern of Wnt5a expression. These results indicate that the effect of Wnt5a on those genes is restricted specifically to the penumbra, not the contralateral side. As Wnt5a is available in both hemispheres, stress is required for Wnt5a to act as an inflammatory mediator. Overall, these results point toward Wnt5a acting as a non-conventional inflammation mediator and DHA/NPD1 signaling acting as a regulatory mechanism that specifically switches off Wnt5a-triggered gene expression and Wnt5a extracellular availability.

## Discussion

Wnt5a fosters neuronal survival by negatively regulating the cell-cycle (Zhou et al. 2017), protects against neurodegeneration through glucose metabolism promotion (Cisternas et al. 2016), and displays a high abundance that correlates with cancer aggressiveness (Binda et al. 2017). We found that several forms of Wnt5a are released by human RPE cells and that deglycosylation allows visualization of Wnt5a (Fig. 1h and Supplementary Fig. S3). We are currently exploring whether glycosylation is a cellular regulatory mechanism of Wnt5a extracellular availability. In addition, the context in which Wnt5a targets a cell also determines its activity. By characterizing the vesicles containing FZD_5_/Wnt5a, FZD_5_/clathrin, or Wnt5a/clathrin, we defined that the variation does not always follow the same pattern for the three types of vesicles (Fig. 2f and Supplementary Fig. S5), indicating that other receptors are involved in the inflammatory signaling of hpRPE cells. It is well-known that Wnt5a also triggers the internalization of FZD_4_ (Chen et al. 2003), though whether or not this pathway is conducive to the activation of NFkB is unclear. In hpRPE cells, FZD_4_ and FZD_8_ are not expressed (Supplementary Fig. S11), so we can discard its contribution. Because Wnt5a may act as ligand of more than one receptor, the coexistence of those receptors in one cell may determine that one wnt ligand triggers concurrently different signaling pathways. In this scenario, the result effect will be determined by the integration of the collective reactions elicited. Here we noticed a possible contribution of FZD_2_ and FZD_7_ to the activation of NFkB via Wnt5a (Fig. 3d); however, the combination of the reduced number of p65-positive nuclei and the low content of the aforementioned transcription factor means that it is very likely that the main pathway was determined by FZD_5_ and ROR_2_. We are currently investigating the other two Frizzled receptors, which will be the subject of future studies.

The presence of Wnt5a enhanced apoptosis beyond the levels induced by UOS, resembling TNFα action (Fig. 1d, Fig. 3g, and Supplementary Fig. S1c), whereas the sole presence of Wnt5a did not trigger RPE cell death (Fig. 1d, Fig. 3g, and Supplementary Fig. S1c). Thus, within cells in a susceptible state, Wnt5a affects their fate since they might be more likely to succumb to initial insults, such as UOS, adding another layer of regulation to the Wnt ligand function. Insults and exposure to Wnt5a enhance susceptibility to the containment of cell integrity and survival.

**Fig. 6:**
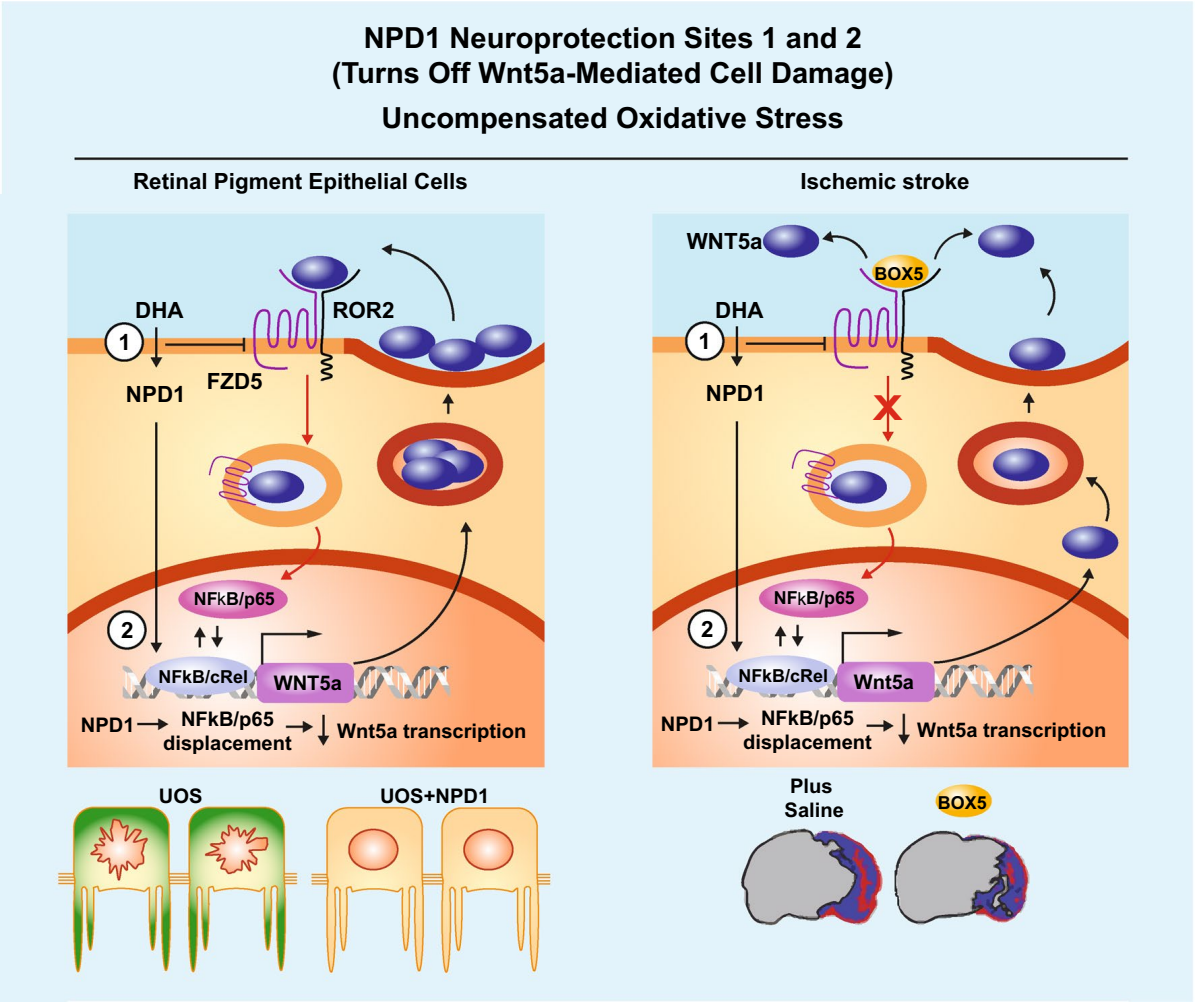
NPD1 Neuroprotection sites model. In retina pigment epithelial cells (left panel), the evidence gathered in the present manuscript points to a Wnt5a-positive feedback loop consisting of the initiation of the signaling; when Wnt5a interacts with FZD5 and ROR2, it is internalized via clathrin-mediated endocytosis, and the process elicits the activation of NFkB/p65 in cells undergoing oxidative stress conditions. NPD1 interferes with this amplification of the inflammatory signaling at two sites: 1) by reducing the availability of FZD5 (Fig. 1e, g) and thus the internalization of Wnt5a and downstream events, and 2) by increasing the availability of cREL that competes with p65 in the dimeric NFkB and decrease the transcription of Wnt5a (Fig. 4). In ischemic stroke (right panel), the reperfusion after the ischemic episode induces an oxygen-rich environment, similar to uncompensated oxidative stress status. Here, the addition of box5, a hexapeptide that competes for the binding of Wnt5a to the receptor, demonstrated that wnt5a inflammatory loop occurs under these conditions, and it is responsible for at least part of the damage observed in ischemic stroke. DHA, known to be converted into NPD1 and for activating cREL in rat brain (Calandria et al. 2015), reduced the initial increase of Wnt5a messenger in brain tissue (Fig. 5f, i) and circulated Wnt5a protein (Fig. 5g). Treatment with DHA also induced a steep decrease in proinflammatory cytokines, and SASP-related expression downstream of the Wnt5a signaling loop was in agreement with the model established in RPE cells

Wnt ligands are secreted via exosomes (Gross et al. 2012), but Wnt5a was not in the exosomal form in hpRPE cells when we extracted them by ultracentrifugation (Fig. 1j) and by differential solubility (data not shown). Only by precipitation of 100,000 rpm supernatant did we rescue the sWnt5a (Fig. 1j). Therefore, in RPE cells under UOS, our data show that even Wnt5a is trafficked via vesicles (Fig. 2, Supplementary Fig. S8, and Fig. 6), and its release is produced mainly in an exosome-free manner (Fig. 1f). Wnt5a signal increases with the addition of recombinant Wnt5a (Fig. 1d, Fig. 3a, Fig. 4e, and Fig. 5b), reflecting the ability of Wnt5a to enhance not only its own expression but also FZD_4_, FZD_2_, and FZD_5_ receptor endocytosis (Chen et al. 2003; Kurayoshi et al. 2007; Shojima et al. 2015). Vesicular Wnt5a was detected in RPE cells (Fig. 2), as was the secretion of mature Wnt5a in vesicles, and Golgi supported the maturation of the protein (Kurayoshi et al. 2007). Therefore, different size vesicles carrying Wnt5a detected in RPE cells (Supplementary Fig. S8) may harness maturation, degradation, and sorting of Wnt5a through a dynamic, steady-state with controlled extracellular release; transcription is variable depending on UOS (Fig. 1c and Fig. 4c). CME-inhibitor, Pitstop2, interrupted Wnt5a endocytosis in resting cells and in NPD1-treated cells, ensuing in an increase in sWnt5a under these conditions (Fig. 3i) and pointing to the role of NPD1 in the fate of secreted Wnt5a.

As a macrophage activator in the non-sterile innate response (Pereira et al. 2008), Wnt5a also mediates non-sterile inflammatory responses in non-immune cells (Zhao et al. 2014). Our current results indicate that non-immune cells evoke the inflammatory response in sterile conditions. The signaling pathways involved in RPE cell damage and in stroke display similarities in the stroke penumbra, Wnt5a synthesis increases (Fig. 5f, i) in the ipsilateral side (Fig. 5f), Wnt5a protein elevation in both sides (Fig. 5i) and presence in the blood plasma after stroke, providing a potential stroke biomarker candidate.

As macrophage infiltration occurs during stroke (Lu et al. 2021), the increase in Wnt5a more than likely enhances the destructive activity of these cells in the brain. Moreover, systemic Wnt5a may be involved in the recruiting and stimulation of innate immune cells since Wnt5a activates microglia, dendritic cells, and macrophages (Shimizu et al. 2016), enhancing other pathways such as non-canonical Wnt and TLR-triggered signaling. DHA treatment brought down bloodstream Wnt5a protein after 24 h of stroke onset (Fig. 5g). This was an early event considering that at 3–7 days after stroke, the levels of Wnt5a in blood decreased. Box5 administration did not affect Wnt5a abundance as DHA did, implying that Box5 and DHA act differently. We propose that DHA conversion into NPD1 activates cRel expression (Calandria et al. 2015), which in turn halts Wnt5a transcription, release, and autocrine and paracrine binding to FZD_5_, while Box5 impedes the latter. Our data show Box5 and DHA protection at the neurological/behavioral level as well as by reduction of stroke damage (Fig. 5c-e); however, DHA seems to produce a sustained improvement. The source of circulating Wnt5a is unknown and currently under investigation. There are at least two possible sources of secreted Wnt5a: blood cells such as monocytes (Sessa et al. 2016) or endothelial cells that produce the Wnt ligand in certain conditions, inducing permeabilization and angiogenesis (Korn et al. 2014; Skaria et al. 2017). The elevation in Wnt5a found in the contralateral hemisphere (Fig. 5f) may be explained by damage or permeabilized brain blood barrier that allows the entrance of the circulating Wnt ligand. Alternatively, without increasing its transcription, the hike in the contralateral Wnt5a protein may be explained by glutamate excitotoxicity in the contralateral area to a lesser degree than in the ipsilateral side (Li et al. 2012). Finally, gene expression linked to Wnt5a, UOS, and inflammation via NFkB/p65 or Wnt signaling increased markedly in the ipsilateral but not in the contralateral side, and such an increase was counteracted by DHA, as indicated by the gene expression profile (Fig. 5j).

Transcellular inflammation signaling is not well-understood. Here, we propose that non-immune cells under UOS conditions are capable of transferring inflammatory signals that may only affect susceptible cells and lead to their damage (Fig. 6). NPD1 interferes with the Wnt5a feedback loop at two strategic signaling points, promoting cell survival in bystander cells. The ischemic stroke model provided an in vivo test of our observations at the RPE cell level. We have previously found that this lipid mediator synthesis correlated with cRel, a member of NFkB/p65, activity in RPE cells and in post-stroke penumbra (Calandria et al. 2015). We demonstrate that cRel binds to at least two regions in the Wnt5a promoter with high affinity (Fig. 4d-f and Supplementary Table S4). cRel overexpression suppresses the Wnt5a transcription in response to UOS, probably displacing those NFkB dimers containing p65 (Fig. 3). Post-stroke, we observed a similar trend seen in the RPE: Wnt5a transcription was elevated at 1 and 3 days after stroke only in penumbra at the ipsilateral hemisphere, while the expression in the contralateral side was not affected (Fig. 5f). These results propose that only susceptible cells affected directly by ischemia–reperfusion promote the Wnt5a-positive feedback loop at the transcriptional level. DHA enhances NPD1 synthesis and induces translocation of cRel in neurons (Calandria et al. 2015), which prevents p65-driven activation of Wnt5a transcription. Stressors like NMDA glutamate receptor activation trigger transcription-independent Wnt5a translation (Li et al. 2012), which means that Wnt5a transcription and translation may be uncoupled events and may explain some of our observations (Fig. 5f, i).

These findings uncovered a new participant in the transfer of inflammatory signals occurring in the retina and brain under UOS and how endogenous neuroprotection mediators derived from DHA may halt damage to enhance cell survival. Understanding these new neuroprotective cellular and molecular mechanisms will enable the exploration of therapeutic avenues to target the onset and early progression of brain and retina damage that include neurodegenerative diseases.

## Materials and Methods

### Experimental Model and Subject Detail

#### Transient Middle Cerebral Artery Occlusion (MCAo)

Animals were housed and treated in compliance with LSU Health Sciences Center Institutional Animal Care and Use Committee (IACUC) protocols. The right MCA was occluded for 2 h by intraluminal filament, as we described previously (Belayev et al. 2011). Briefly, the right common carotid artery (CCA) and external carotid artery (ECA) were exposed through midline neck incision and then completely isolated from the surrounding nerves. The occipital branches of the ECA and pterygopalatine artery were ligated. Four cm of 3–0 nylon filament coated with poly-L-lysine was advanced to the origin of MCA through the proximal ECA via the internal carotid artery. The filament was inserted 20 to 22 mm from the bifurcation of the CCA, according to the animal’s body weight. The neck incision was then closed, and the rats were returned to their cages. After 2 h of MCAo, the rats were re-anesthetized with the same anesthetic combination, and the intraluminal filament was gently removed. The animals were allowed to survive at different times, according to the experimental protocol, with free access to water and food.

### Behavioral Tests

Behavioral tests were conducted before, during MCAo (at 60 min), and then at 24 h, 48 h, 72 h, or 7 days after MCAo by an investigator blinded to the experimental groups. The battery consisted of two tests: (1) postural reflex to examine the upper body posture when the rat was suspended by tail, and (2) forelimb placing test to assess the forelimb placing responses to visual, tactile, and proprioceptive stimuli (Belayev et al. 2011). Neurologic function was graded on a scale of 0 to 12 (normal = 0, maximal deficits = 12), as we described previously (Belayev et al. 2011). The severity of stroke injury was assessed by behavioral examination of each rat at 60 min after onset of MCAo. Rats that did not demonstrate high-grade contralateral deficit (score, 10–11) were excluded from further study. Two animals were excluded for this reason.

### Treatment Groups

Docosahexaenoic acid (DHA; 5 mg/kg, Cayman, Ann Arbor, MI, USA) or vehicle (0.9% saline) was administered intravenously into the femoral vein at a constant rate over 3 min using an infusion pump at 3 h after onset of MCAo. For the western blot study, rats were sacrificed on days 1, 3, or 7; for real-time PCR study, rats were sacrificed on days 1, 2, or 3.

### Cell Culture, Treatments, and Transfection

Primary human RPE cultures were obtained from human eyecups, provided by the National Disease Research Interchange (NDRI), as described previously (Calandria et al., 2012). hRPE cells were grown and maintained in high-glucose MEM (Life Technologies Corporation) supplemented with 10% FBS (Tissue Culture Biologicals, Inc.), 5% NCS, non-essential amino acids, Penicillin–Streptomycin (100 U/mL), human fibroblast growth factor (FGF) 10 ng/mL and incubated at 37 °C with a constant supply of 5% CO_2_. ARPE-19 cells were plated and grown in DMEM/F-12 containing 10% FBS and 1X penicillin/streptomycin at 37 °C, 5% CO_2_, and 99% relative humidity for 24 h. Silenced 15-LOX-1 cells, described in detail elsewhere (Calandria et al. 2009), are derived from ARPE-19 by stably silencing 15-LOX-1. 15-LOX-1 deficient cells were maintained in the same medium as ARPE-19 with the addition of 500 μg/mL Geneticin (Life Technologies Corporation). Transfection was performed in all cases using Lipofectamine 2000 (Life Technologies Corporation) following the manufacturer’s recommendations. Transfection efficiency was assessed using an expression plasmid-carrying GFP ORF or by including a negative control siRNA conjugated with Alexa Fluor® 488 or 546 (QIAGEN). siRNA efficiency was assessed using SYBR green-based real-time PCR using the primers of Supplementary Table S4 and the percentage of silencing achieved (Supplementary Fig. S9 and Supplementary Fig. S10). Experiments with transfection efficiency that yielded less than 80% were discarded. For siRNA, we used 6 µl of Lipofectamine 2000 per 50 pmol of siRNA per mL of incubation medium, and for plasmids, we used 6 µl of Lipofectamine 2000 per 2 µg of plasmid per mL of the incubation medium. Cells were incubated for 5 h at 37 °C with 5% CO_2_ and 99% relative humidity. After the medium was changed, the cells were left to recover for 24 h before starting treatments. When siRNA/plasmid co-transfection was required, siRNA and plasmid-carrying transfection mixes were prepared separately and added one after the other in the same well. UOS treatments were performed using 600 (ARPE-19) or 1600 (hRPE) µM H_2_O_2_, alone or with 10 ng/mL TNFα only when it is indicated, for the required time indicated in each experiment (these concentrations were previously tested to achieve over 50% apoptosis on ARPE-19 and hpRPE cells). The treatment we used employed the low-serum (0.5% serum) without hFGF. Treatment to induce UOS was performed after 8 h of low-serum medium incubation unless specifically noted. Wnt5a (50 ng/ml), Wnt3a (50 ng/ml) or NPD1 (100 nM) was added immediately before induction of UOS. In the secretion experiments, Pitstop 2 was added to the medium 2 h before harvesting.

### Immunocytochemistry and Hoechst Staining

Immunostaining was performed in 8-well slide chambers and the Hoechst staining in 24-well plates. To carry out immunocytochemistry, a previously described protocol was followed (Calandria et al., 2012). Briefly, cells were fixed using 4% paraformaldehyde in PBS 1X for 20 min at room temperature or overnight at 4ºC. After three washes with PBS, cells were permeabilized using 0.1% Triton™ X-100 for five minutes and blocked using 10% normal serum and 1% BSA for one hour. Primary antibody incubation took place overnight at 4 °C and secondary antibody coupled to Alexa Fluor® 488 and 594 were used to detect Wnt5a and FZD5, respectively. Hoechst staining was performed using same protocol of fixation, but permeabilization was done using methanol for 20 min at room temperature. Immediately after 20 ng/mL Hoechst 33,342 (Life Technologies) in PBS 1X was added. Images for Hoechst staining were obtained using Nikon Ti-U inverted fluorescence microscopes with NIS-Elements BR 3.00 software (NIKON Inc, Melville, NY, USA) and ICC images from Olympus FV1200 and FV3000 confocal with Fluoview software FV10-ASW Version 04.02.02.09 and FV30-ASW (Olympus Corp Center Valley, PA, USA). The Apoptosis analysis was performed using ImageJ 1.48 (National Institutes of Health) and MetaMorph software from Meta Imaging Series 7.8.0.0 (Molecular Devices, LLC, San Jose, CA). Dying cells were identified by size (0 to 50 pixels), intensity threshold and circularity. The ratio of hyperpyknotic cells over the total was calculated for nine randomly chosen fields per sample obtained from three independent wells per experiment. Each experiment was repeated at least three times to confirm the findings. In the experiments involving the early stages of apoptosis, we assessed the total number of cells per field and counted them to ensure unbiased observations. Colocalization of Wnt5a and FZD5 was performed using BioImageXD (Kankaanpää et al. 2012) and IMARIS 9.8 (Bitplane, Oxford instruments, Belfast, UK) on z-stack images obtained at 20X magnification in an Olympus FluoView1200 and FluoView3000 confocal laser-scanning microscopes. Settings were adjusted in the conditions that showed higher intensity and were used throughout the remaining samples. Pearson’s colocalization coefficient (PCC) was obtained using FV1200 analysis software for every field in each experiment, and the mean and SEM are depicted in Supplementary Fig. S8. Colocalized objects and plots of pixels vs. intensity were obtained using ImageJ (Schneider et al. 2012). IMARIS software analysis was performed by building a colocalization channel using the Costes thresholding, and the colocalized elements were analyzed using the spots function using the settings of different spot sites, background subtraction, and local contrast. The output obtained consisted on a collection of sizes (one per element in each picture) or the mean of the intensity in each spot. The data were plotted and analyzed using GraphPad 9 (Prism, San Diego, CA).

### Protein Precipitation and Western Blot

When secretion of Wnt5a was assessed, 1 mL of medium was collected, centrifuged at 13,000 rpm/5 min at 4ºC to remove cell debris and precipitated using methanol/chloroform (Wessel and Flügge 1984). The pellet was resuspended and denatured in 100 µl of 2X Laemmli sample buffer (Bio-Rad Laboratories) at 95 °C for 5 min. MCAo brain tissue and cell samples were homogenized using RIPA (Thermo Fisher Scientific) buffer supplemented with protease and phosphatase inhibitor cocktails (Sigma). The amount of 30 µg of total protein was loaded in NuPAGE® Novex® 4–12% Bis–Tris precast gels (Life Technologies Corporation) or Mini-PROTEAN TGX Stain-free gels 4–15% and run at a constant 120 V for 1 h and 20 min or 250 V for 40 min, respectively. Proteins were transferred to µm Nitrocellulose or LF-PVDF membrane using Bio-Rad Trans-Blot® Turbo™ System (Bio-Rad Laboratories). ECL™ Plex Fluorescent Rainbow Markers (GE Healthcare) were used as the ladder for protein’s molecular weight. Membranes were blocked using 5% non-fat dry milk (Bio-Rad Laboratories) in TBS with 1% Tween® 20 (TBST-10X) for 1 h and incubated overnight with primary antibodies. Anti-mouse or anti-rabbit secondary antibodies conjugated with Cy3 or Cy5 were used to visualize the protein of interest. Immunoblots were documented using LAS 4000 imaging system (GE Healthcare Life Sciences) or ChemiDoc MP Imaging System (BioRad Laboratories). A time course exposition was produced in each case to prevent quantification of saturated images. Densitometry data were obtained using ImageQuant™ TL software and XRS Blot Chemi Software (BioRad Laboratories).

### SYBR Green-Based Real-Time PCR

Brain samples were homogenized on ice by Dounce homogenizer and total RNA was extracted by TRIzol Reagent (Life Technologies Corporation). Cell samples, total RNA was extracted by RNeasy Mini Kit (QIAGEN) following manufacturer’s protocol. The purity and concentration of RNA were determined by NanoDrop ND-1000 Spectrophotometer (Thermo Fisher Scientific). cDNA first strand was obtained from one microgram of total RNA using iScript™ Reverse Transcription Supermix (Bio-Rad Laboratories, CA, USA). The resulting cDNA was used as template SYBR-green or Eva-green-based real-time PCR quantification using SsoAdvance Universal Supermix (Bio-Rad Laboratories). Data were collected and analyzed using CFX Manager 3.0 software, the ΔΔCt method. Melting curve was produced for every run to assure a unique amplified product per primer set. Primers are depicted in Supplementary Table S4.

### Luciferase Assay

For activation of canonical NF-ĸB, cells were co-transfected with plasmid p65/p50 promoter consensus sequence Cignal NF-ĸB Reporter Kit, (QIAGEN) along with both positive (constitutively active promoter) and negative controls and GFP using Lipofectamine 2000 (Life Technologies) following company protocols. β-catenin activity was measured using TOP Flash/FOP Flash constructs obtained from Addgene (Cambridge, MA, USA) (Veeman et al. 2003). Except TOP/FOP flash, the Luciferase activity was standardized using a construct expressing GFP constitutively under CMV virus promoter. To assess luciferase activity, cell lysates were obtained using Passive Lysis Buffer (Promega) and mixed with a luciferase assay reagent (Promega). Chemiluminescence produced by luciferase and fluorescence from GFP was detected using Appliscan 2.3. Data were analyzed using SkanIt 2.3 (Thermo Fisher Scientific).

#### Detection of DNA Binding Motifs and Methylation

To analyze the consensus binding sequences of the Wnt5a promoter A (Katula, et al., 2012), two searching engines were used: TRED (http://rulai.cshl.edu/TRED), which uses the JASPAR database, and TFBind (http://tfbind.hgc.jp/) that uses TRASFAC database (Tsunoda and Takagi 1999; Jiang et al. 2007). Supplementary Table S1 contains scores and positions obtained for the 7 regions identified that cREL potentially binds (Fig. 2). Analysis of the methylation was performed using MethPrimer (http://www.urogene.org/methprimer2/) (Li and Dahiya 2002).

### ChIP Assay

The chromatin immunoprecipitation assay was performed using SimpleChIP® Plus Enzymatic Chromatin IP Kit (Cell Signaling Technology, Boston, MA, USA) following the manufacturer’s recommendations including ChIP validated cREL antibody. Positive control Histone H3 antibody and normal serum was provided by the kit, as well as control primers for human RPL30 Exon 3. Primers A1 to 4 were designed using Primer-Blast at NCBI platform (Ye et al., 2012) and are depicted in Supplementary Table S5. The immunoprecipitated samples real-time PCR values were standardized using 10% of the input chromatin preparation using primers A1, A2, and A3 pooled values and RPL30 Exon 3 values.

#### Quantification and Statistical Analysis

Data are presented as histograms with mean values ± SD or Boxplot, which depict median quartiles 1, 3, and maximum and minimum observations. Repeated measures analysis of variance (ANOVA) one or two ways followed by Bonferroni procedures to correct for multiple comparisons was used for intergroup comparisons. The samples were tested for Normality and Homoscedasticity prior application of ANOVA. Multiple comparisons were performed using Tukey’s Honest Significant Difference. Plotting and statistical analysis were performed using Excel 2016 or analyzed using GraphPad 9 (Prism, San Diego, CA). Differences at P < 0.05 were considered statistically significant.

## Supplementary Information

Below is the link to the electronic supplementary material.

Supplementary file1 (DOCX 2817 kb)

## Data Availability

There are no restrictions on materials. All data are available in the main text or the supplementary materials.
