# Supplementary material for: cRel and Wnt5a/Frizzled 5 Receptor-Mediated Inflammatory Regulation Reveal Novel Neuroprotectin D1 Targets for Neuroprotection
Source: Cell Mol Neurobiol. 2022 May 27;43(3):1077–96. doi: 10.1007/s10571-022-01231-6 (PMC10006067; doi:10.1007/s10571-022-01231-6)
Supplement: Supplementary file 1 — Supplementary file1 (DOCX 2817 kb) [file 10571_2022_1231_MOESM1_ESM.docx]

Supplementary Materials for

**cRel and Wnt5a/Frizzled 5 receptor-mediated inflammatory regulation reveal novel Neuroprotectin D1 targets for neuroprotection**

Jorgelina M. Calandria, Khanh V. Do, Sayantani Kala-Bhattacharjee, Andre Obenaus, Ludmila Belayev, and Nicolas G. Bazan*

*Corresponding author. Email: [nbazan@lsuhsc.edu](mailto:nbazan@lsuhsc.edu)

**This PDF file includes:**

Supplementary Figs. 1 to 14

Supplementary Tables 1 to 5

References (1 to 10)


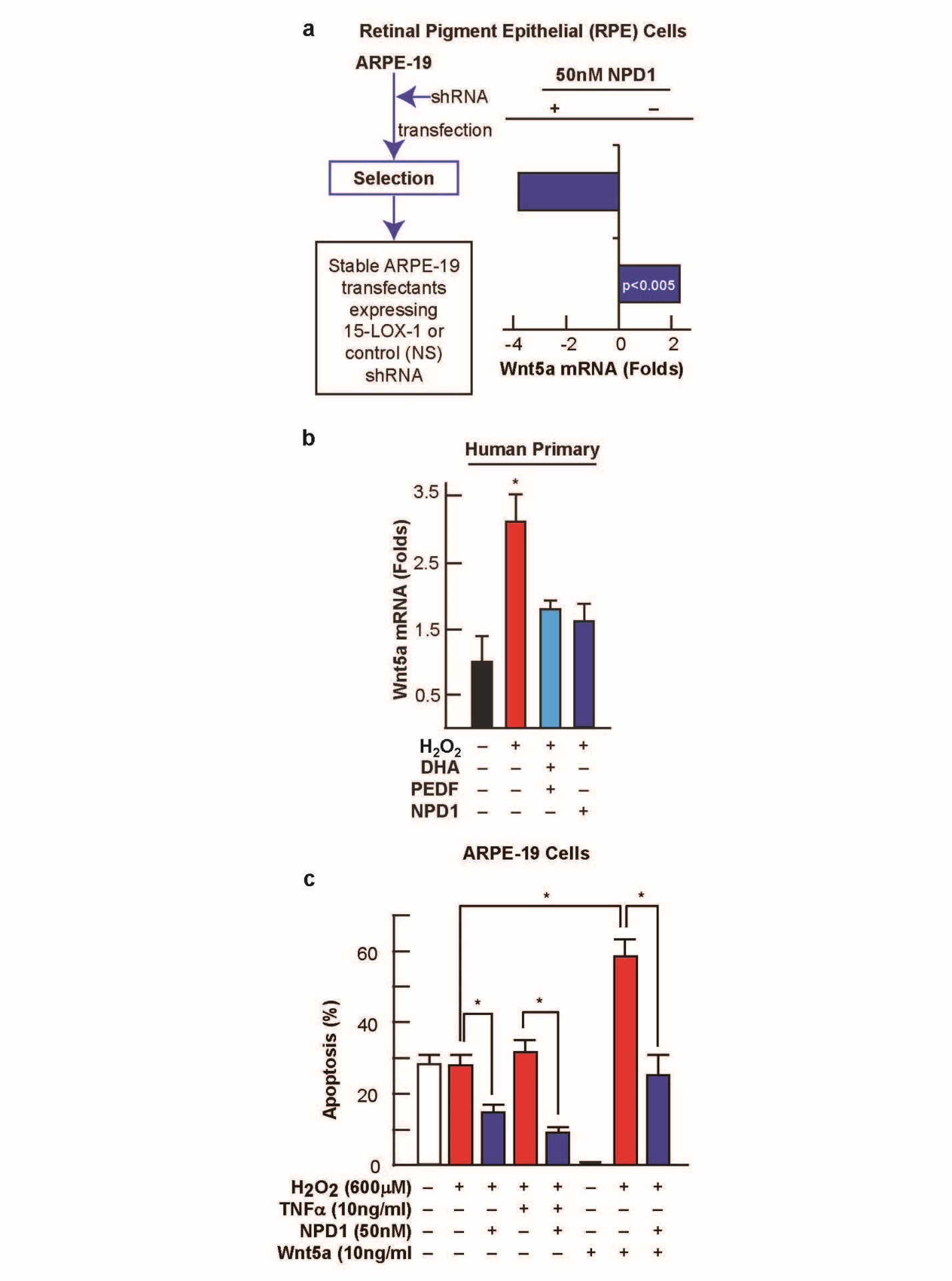


**Supplementary Fig. 1. Wnt5a was upregulated in ARPE-19 cells deficient in 15-LOX-1 undergoing UOS and downregulated by NPD1.** (**a**) Design for experiments leading to the identification of Wnt5a. Uncompensated oxidative stress (UOS)-triggered an increase in Wnt5a expression was reversed by NPD1 in 15-LOX-1d. 15-LOX-1d cells, which show depletion in NPD1 synthesis (*1*), were used to determine genes regulated by the lipid messenger in a microarray assay in ARPE-19 cell 600µM H_2_O_2_ plus 10 ng/ml TNFα was used. Representative values of three independent experiments. ANOVA and test for false positives were applied to select regulated genes on microarray output. (**b**) Quantification of mRNA of Wnt5a in primary human RPE cells. Confirmation of the NPD1-regulation of Wnt5a transcription. 1600 µM H_2_O_2_ was applied to hRPE cells, plus 10 ng/ml TNFα to confirm microarray output using SYBR green-based real-time PCR in human primary cells. Representative values of 3 independent experiments. Bars represent the mean + standard error of the mean of 3 different experimental subjects. (**c**) Wnt5a enhances the percentage of cell death induced by H_2_O_2_ in ARPE-19 cells. Hoechst-positive ARPE-19 cells were beyond H_2_O_2_-induced levels. The criteria used to determine Hoechst-positive cells and the experimental design is depicted in **Fig. 1a**. The bars represent the mean of 3 measurements and the standard error of the mean. *p<0.05.


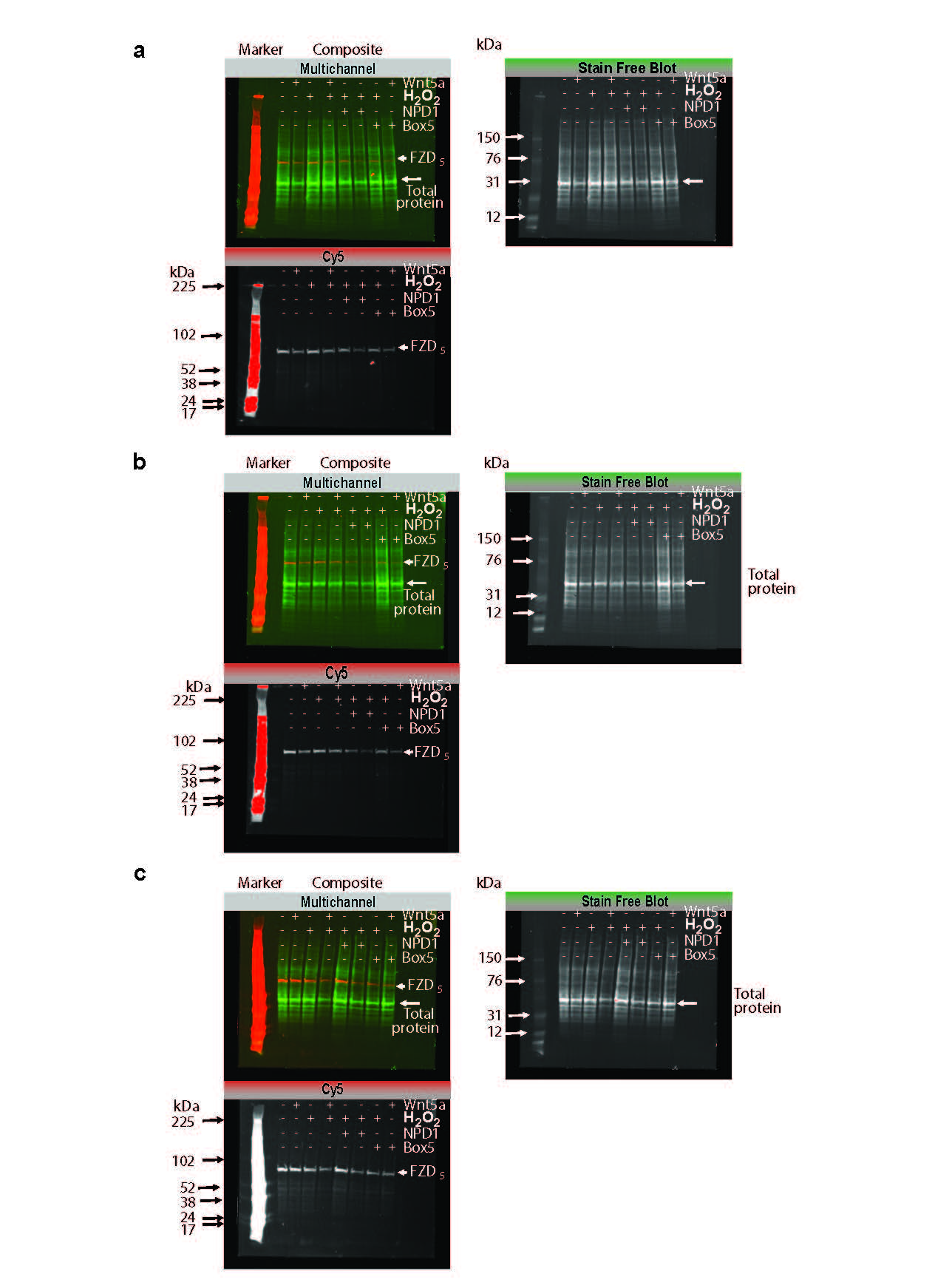


**Supplementary Fig. 2. Whole membranes for the western blots in Fig. 1g.** FZD5 protein was quantified using western blot in hpRPE cells exposed to 1600 µM H_2_O_2_ in the presence or absence of 50 ng/ml Wnt5a, 100 nM NPD1 and/or 100 µM Box5. The level of total protein was measured using stain-free gel blot (BioRad) in the Cy3 channel and used as standardization for the samples.


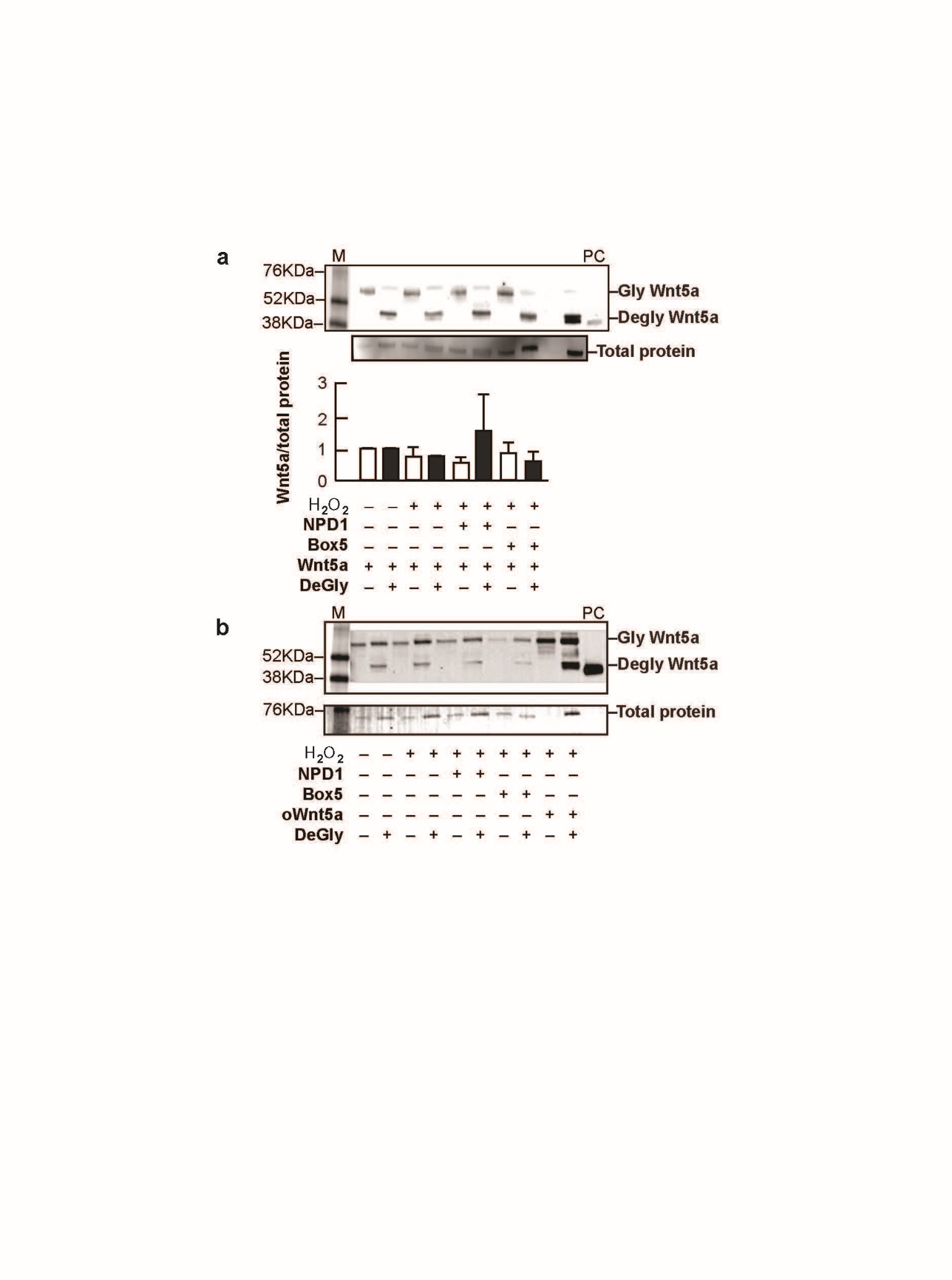


**Supplementary Fig. 3. Deglycosylation of Wnt5a secreted by hpRPE cells.** (**a, b**) Medium from hpRPE cells undergoing UOS was incubated with recombinant Wnt5a (lanes 2-9) in the presence or absence of Box5 or NPD1. Lane 10 and 11, medium from cells overexpressing Wnt5a ORF. Lane 12 = recombinant Wnt5a (R&D). Lane 1 = Rainbow fluorescent marker (GE). The secreted Wnt5a protein was concentrated from the medium by Chloroform/Methanol precipitation. The pellet was resuspended and digested with N and O-glycosylases (Degly). In parallel, non-digested samples (Gly) were run. Western blots were replicated using two different antibodies and a positive control. The antibody used against Wnt5a was the Monoclonal clone # 3D10 from Life Technologies/Thermo Fisher Scientific Cat# MA5-15511 (**a**) and Monoclonal Rat IgG2A Clone # 442625 from R&D cat# MAB645 (**b**). Positive control = Recombinant Human/Mouse Wnt-5a Protein produced in Chinese Hamster Ovary cell line, CHO-derived Wnt-5a protein (Gln38-Lys380).


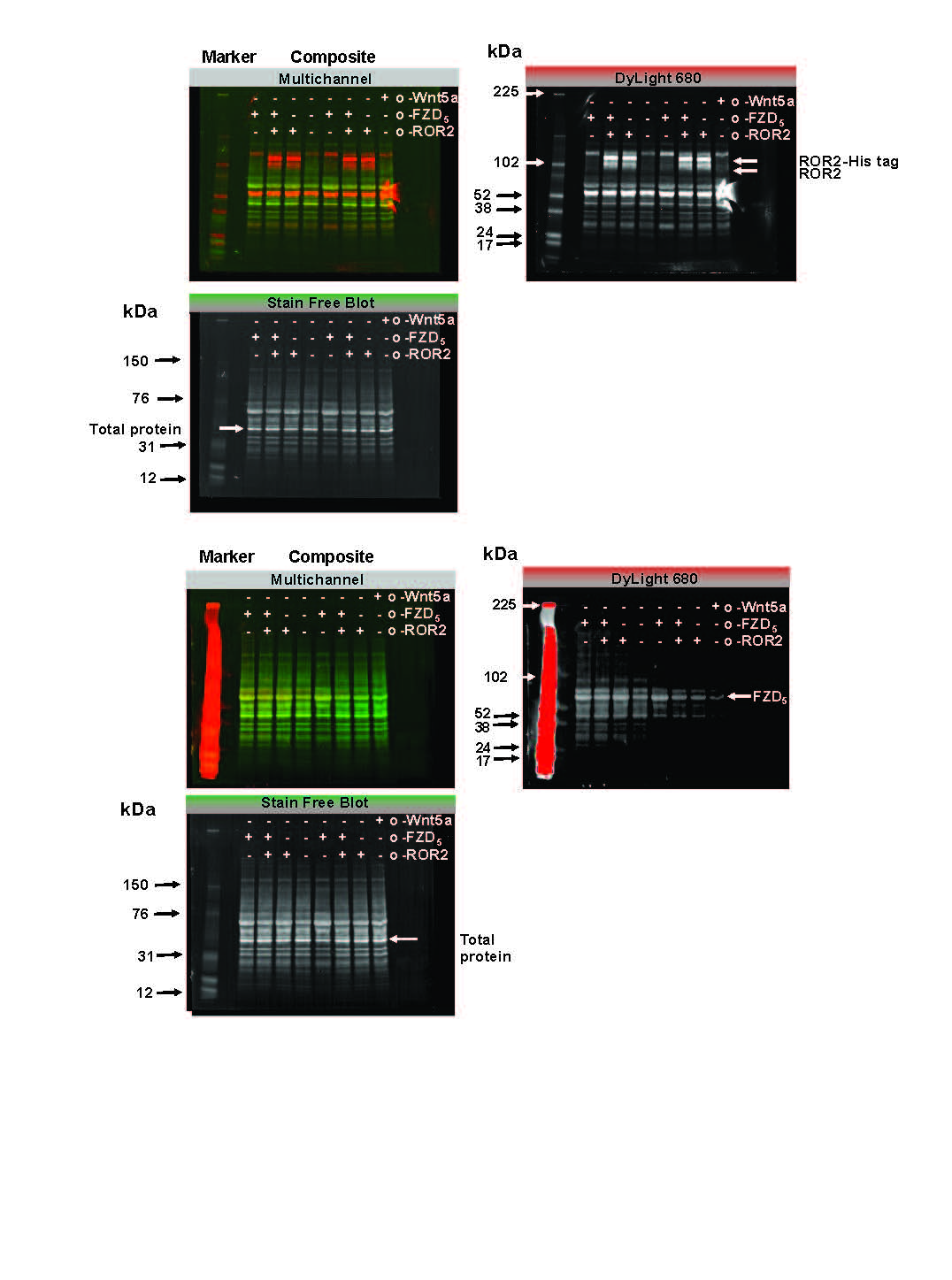


**Supplementary Fig. 4. Whole membranes for the western blots in Fig. 2e.** FZD5 and ROR2 were overexpressed for 48 hours prior to treatment (**Fig. 2f**). Wnt5a was also overexpressed for the same period. The levels of the three proteins were measured by western blot previous at the moment of the treatment initialization of **Fig. 2f** and **Supplementary Fig. 4**. The level of the total protein was measured using stain-free gel blot (BioRad) in the Cy3 channel and used as standardization for the samples.


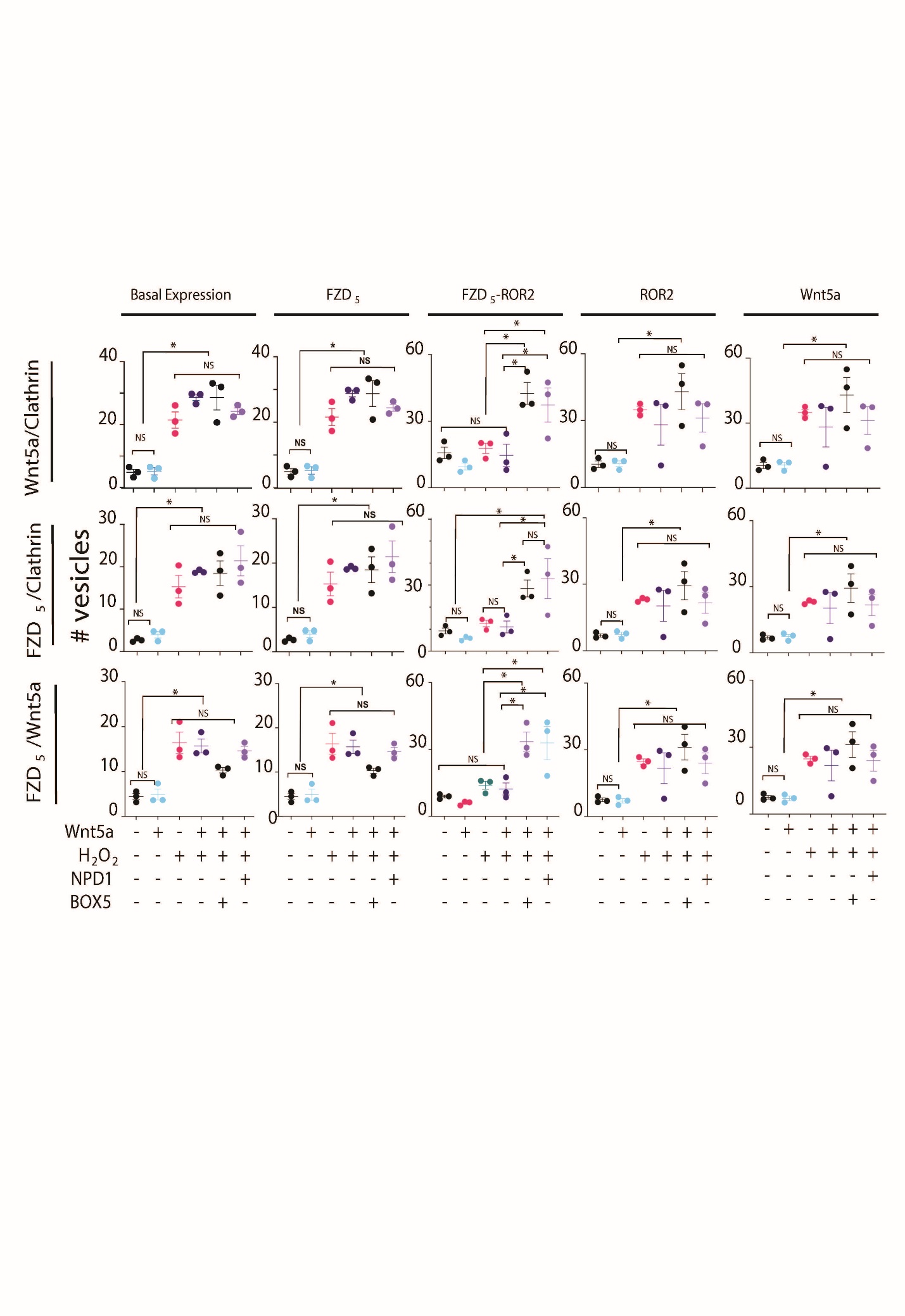


**Supplementary Fig. 5. Number of spots or vesicle corresponding to Fig. 2f.** The hpRPE cells overexpressing FZD_5_ and ROR2, separately or together, Wnt5a or a control vector were treated with 1600 µM H_2_O_2_, 50 ng/ml Wnt5a, 100µM BOX5 or 100 nM NPD1 for 2 hours. Confocal Z-stacks images were analyzed using IMARIS 9.8 spots module, and the number of vesicles (spots) observed by immunocytochemistry targeting Wnt5a, FZD_5_, and Clathrin were plotted. The sum of the intensity for the colocalization of the signal observed for Wnt5a/Clathrin, FZD_5_/Clathrin and FZD_5_/Wnt5a was measured with the Spots module using batch processing (**Fig. 2f**). Each spot corresponds to one individual or clusters of vesicles observed in hpRPE cells under UOS in the presence or absence of NPD1 or Box5 and Wnt5a in the cells expressing the ORFs (open reading frame) tested by WB in (**Fig. 2f and** **Supplementary Fig. 4**).


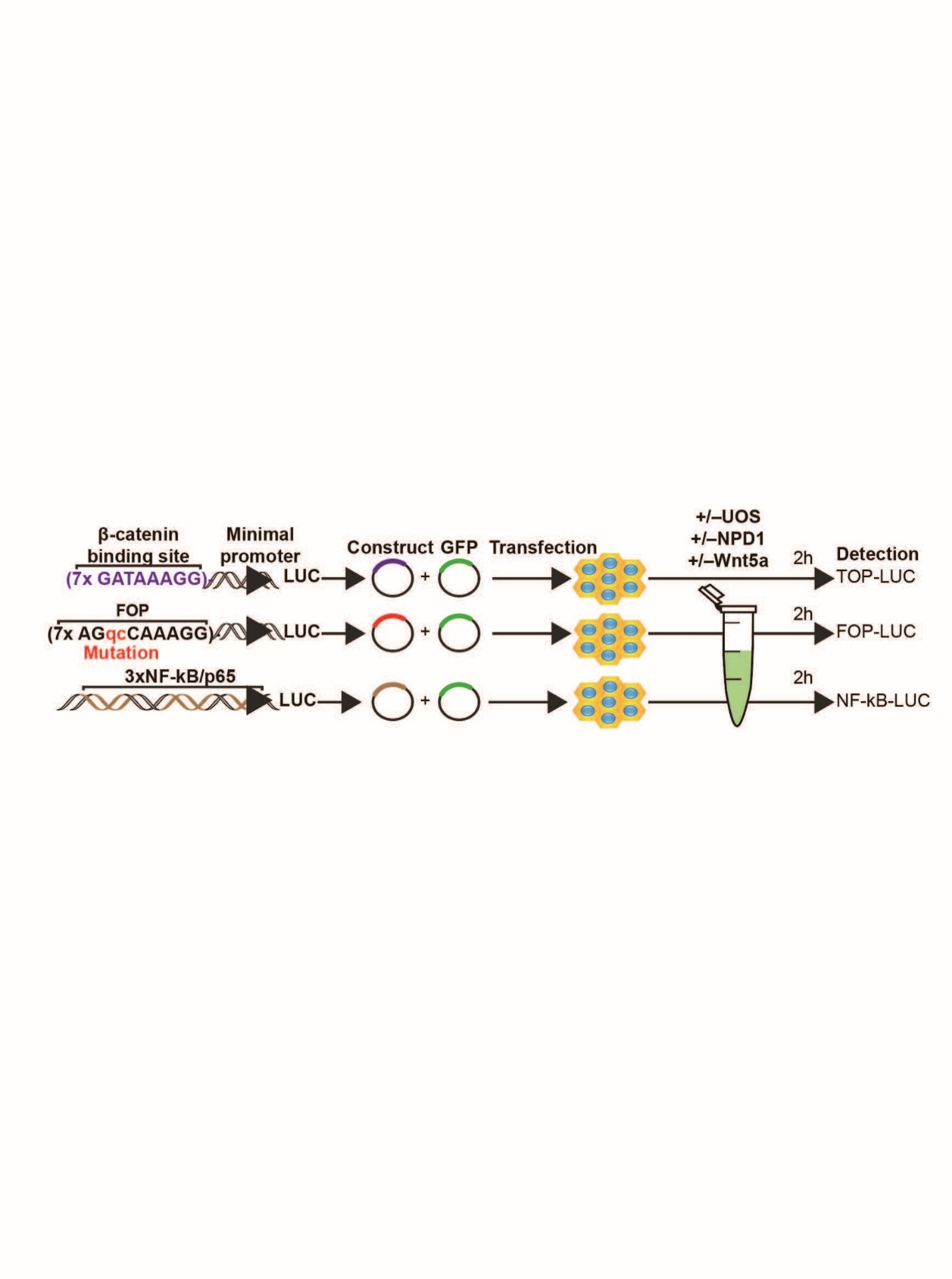


**Supplementary Fig. 6. TOPFlash/FOPFlash and NFkB/p65 reporter constructs.** TOP-Flash (wild type) and FOP-flash (mutated) and three high affinity p65 binding sites arranged in tandem driving the expression of luciferase to detect β-catenin and NFkB activation correspondingly were transfected in hpRPE cells (**Supplementary Fig. 7 and Fig. 3a, i**). The standardization and transfection control was performed by co-transfecting a plasmid that constitutively expressed GFP.


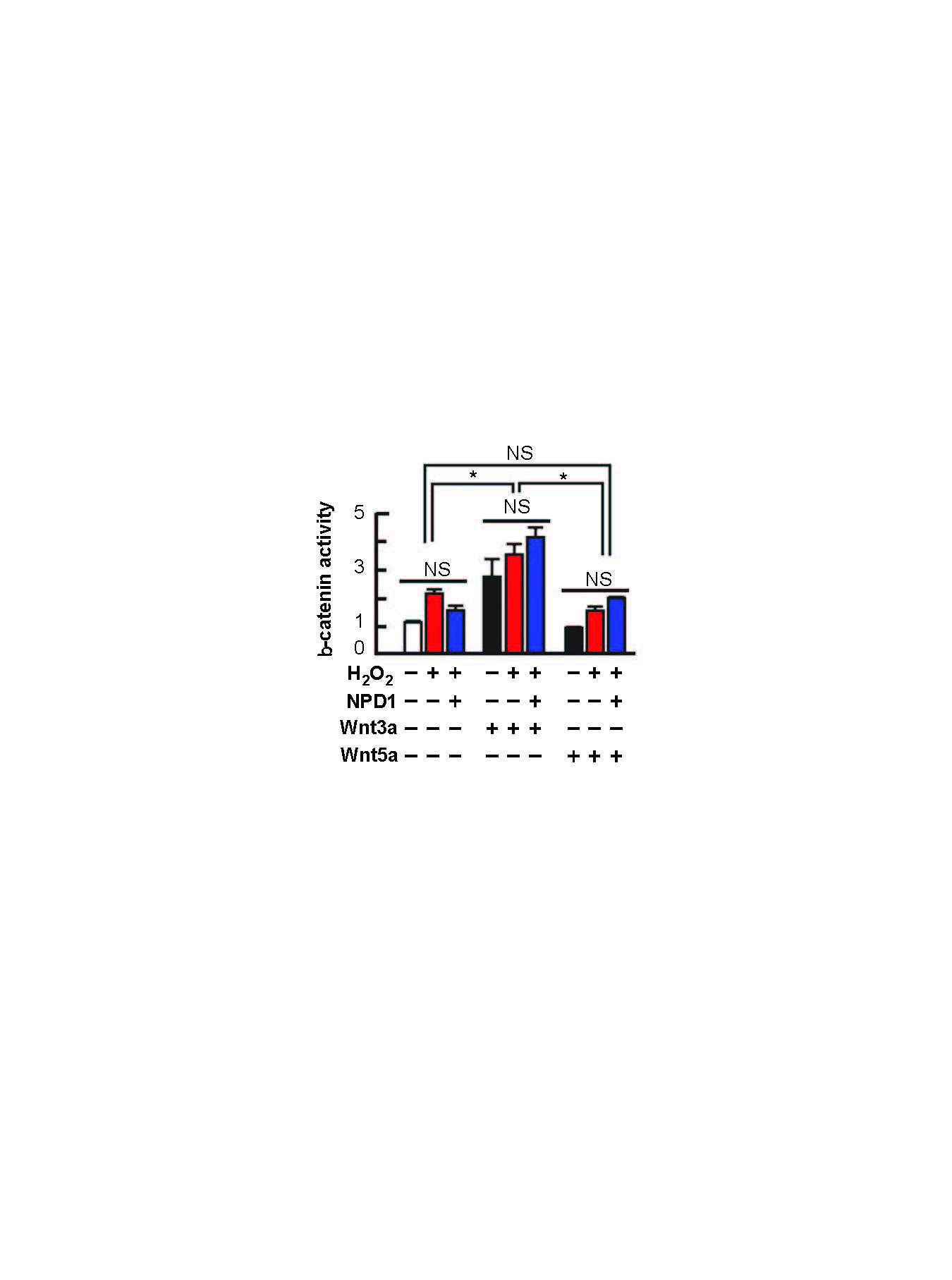


**Supplementary Fig. 7. Luciferase assay using TOP-Flash (wild type) and FOP-flash (mutated) to detect β-catenin activation when Wnt5a was added to the medium of hpRPE cells.** Standardization was made using a plasmid expressing GFP. 1600 µM H_2_O_2_ was used +/- 100nM NPD1 and Wnt5a or Wnt3a. Bars are the mean of three measurements and the standard error of the mean. *p<0.05.


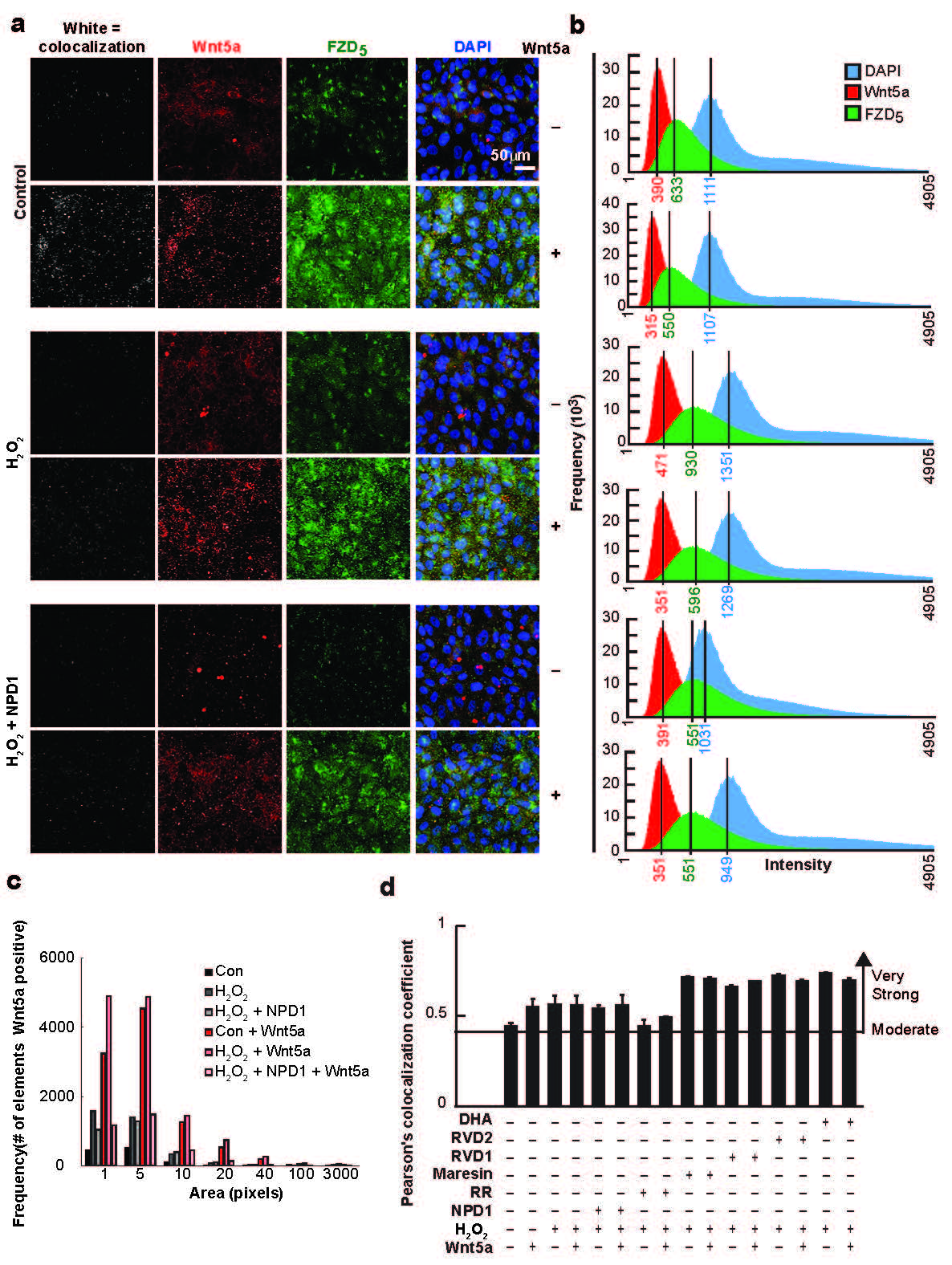


**Supplementary Fig. 8. Colocalization detection of FZD5 and Wnt5a signal using ImageJ.** (**a**) Representative images of colocalized signal of FZD5 and Wnt5a in hRPE cells. Cells were incubated for 2 hours with 1600 µM H2O2 +/- 100nM NPD1 and 50 ng/ml Wnt5a. Immunostaining of Wnt5a (Red) and FZD5 (Green) and z-stack images using BioImageXD (20X). Lasers set up using Control + Wnt5a and used without modification to take remaining pictures. Colocalization of the two signals is in the left column (white). (**b**) Total signal intensity of 3 channels (DAPI = blue, Alexa 488 = green and Alexa 594 = red) in 3 random fields/well/condition. On the right of each row, histogram of intensity vs. frequency depicts pixels numbers showing intensity value on X-axis. The upper limit intensity is set at 4095. Black vertical lines for each channel indicate the mode (most frequent observation) to designate the intensity at which each curve reaches its maximum. Signal points or clusters of pixels showing colocalization (A-left column) were quantified using ImageJ of 3-6 random fields encompassing 1 or 2 wells in up to 3 independent experiments. (**c**) Frequency vs. Area histogram for representative fields showing different sizes of clusters of Wnt5a positive signal. (**d**) Pearson’s colocalization coefficient (PCC) for the first experiment in the series of colocalization by immunocytochemistry. The PCC values obtained for all slices of z-stacks of 3 fields were averaged and plotted. Bars represent the mean of 3 measurements and the standard error of the mean. *p<0.05.


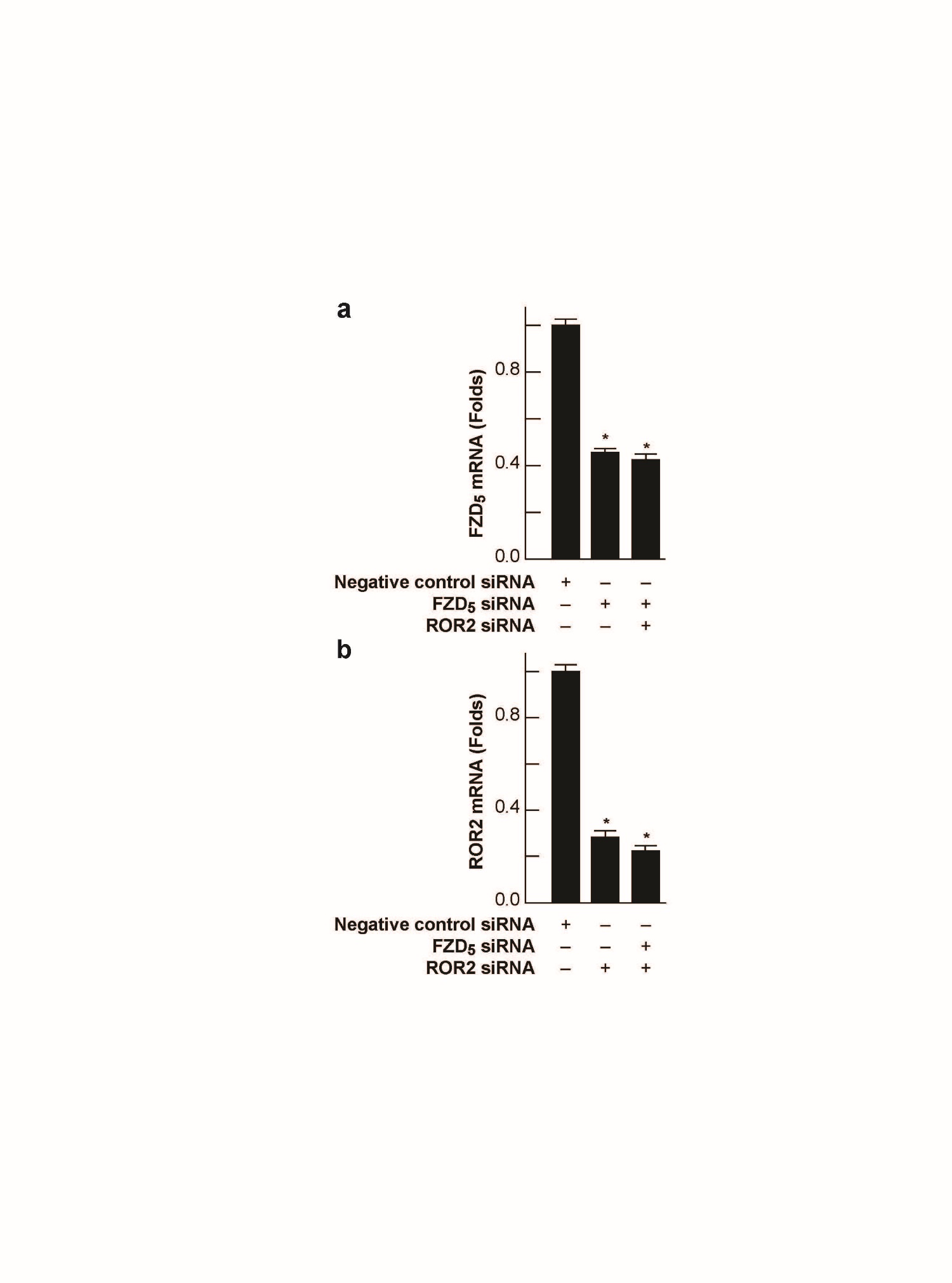


**Supplementary Fig. 9. Quantification of mRNA of FZD_5_ and ROR2 in silenced cells.** (**a**) FZD_5_ and (**b**) ROR2 mRNA quantification on Negative control, FZD_5_, and FZD_5_ plus ROR2 siRNA transfected human RPE cells (**Fig. 3b**). Controls corresponding to experiment. The bars represent the mean of 3 measurements and the standard error of the mean. *p<0.05.


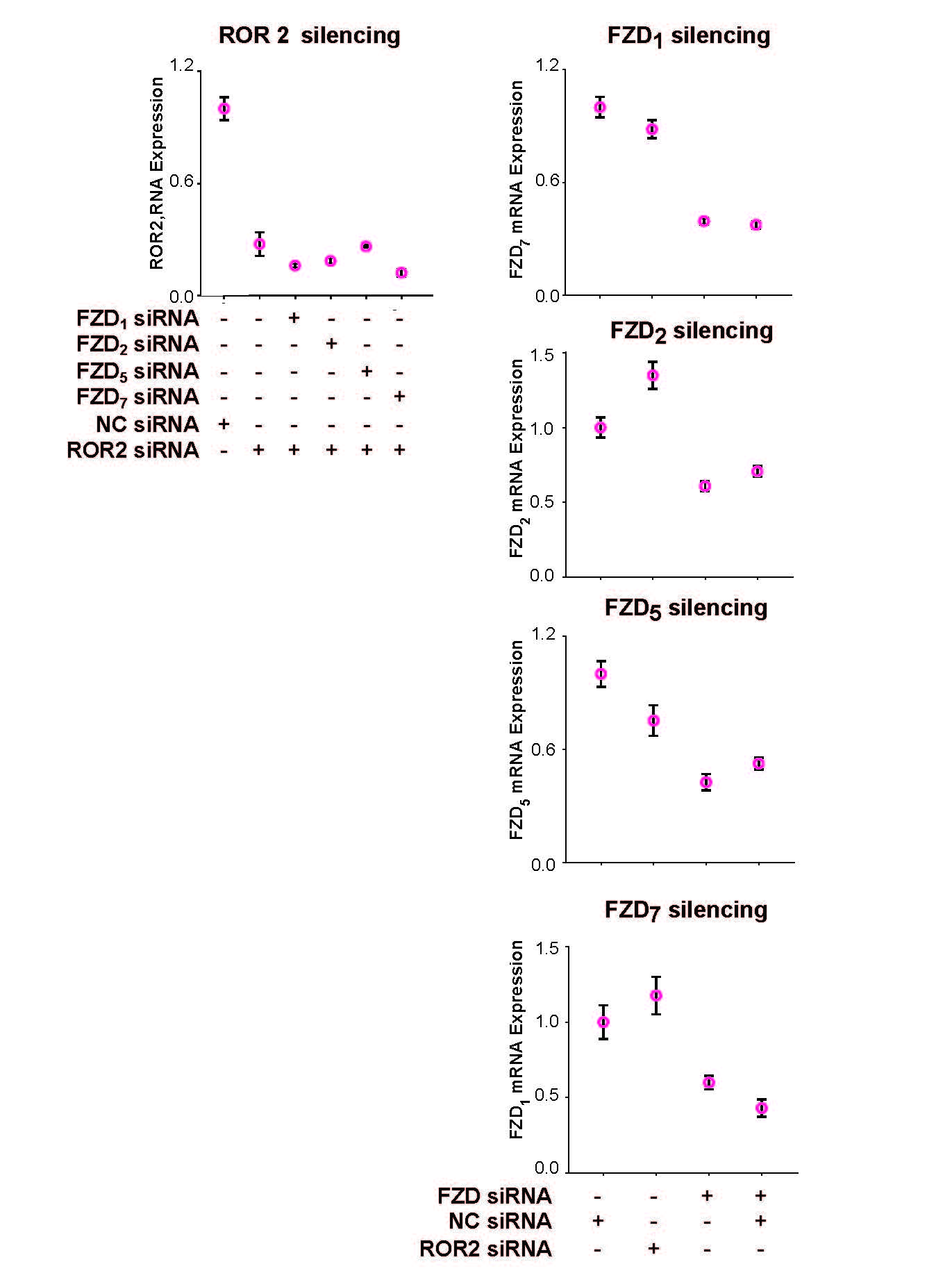


**Supplementary Fig. 10. Quantification of mRNA of FZD_1_, FZD_2_, FZD_5_, FZD_7_, and ROR2 separately and together in silenced cells.** (**a**) FZD_5_ and (**b**) ROR2 mRNA quantification on Negative control, FZD_5_, and FZD_5_ plus ROR2 siRNA transfected human RPE cells (**Fig. 3d**). Controls corresponding to experiment. The bars represent the mean of three measurements and the standard error of the mean. *p<0.05.


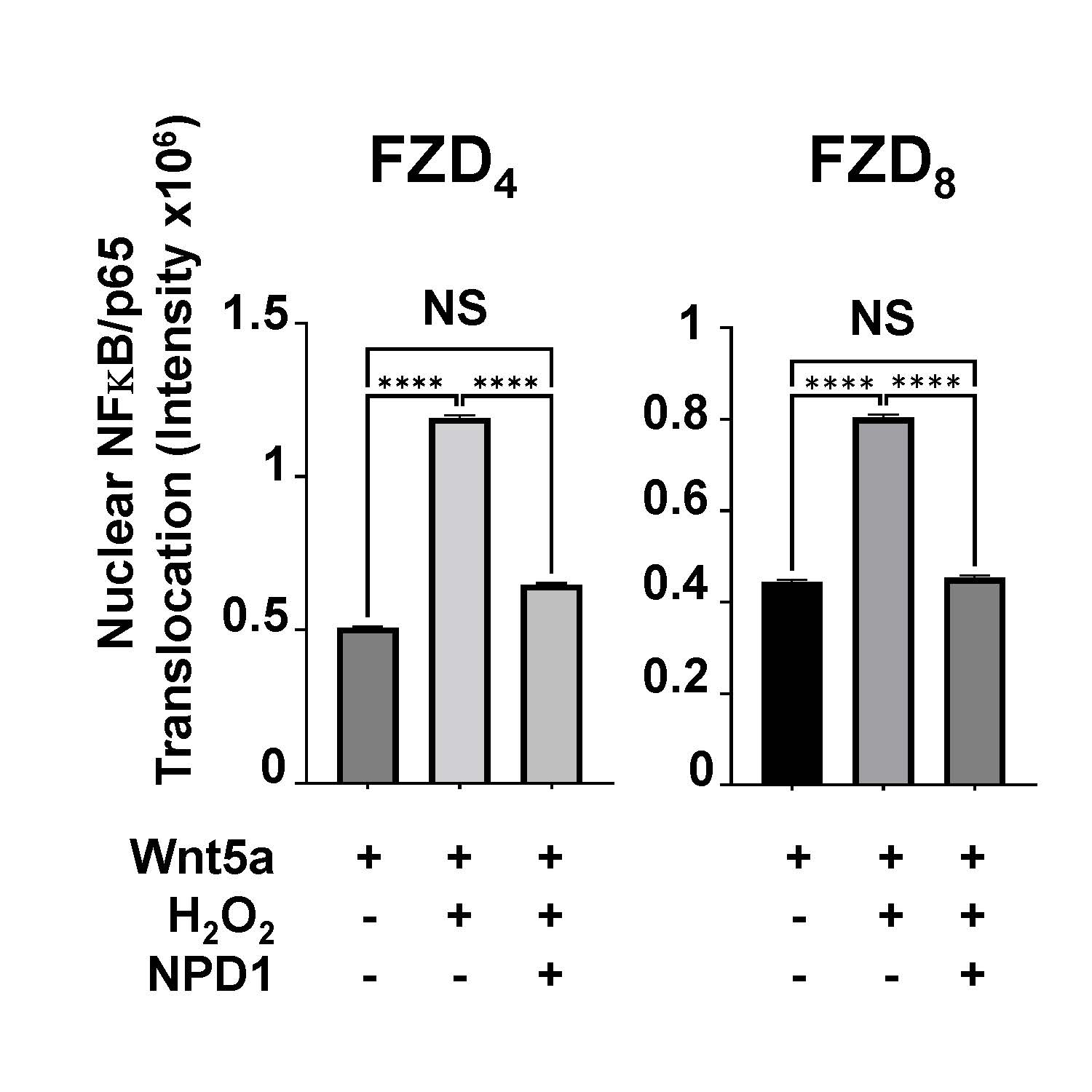


**Supplementary Fig. 11. NFkB nuclear translocation in FZD_4_ and FZD_8_ silenced hpRPE cells.** Silencing of FZD_4_ and FZD_8_ alone in hpRPE cells expressing p65-GFP were treated with 50 ng/ml Wnt5a in the presence or absence of H_2_O_2_ to induce UOS and NPD1 for two hours. Nuclear p65 was assessed by analyzing confocal Z-stack images with Imaris 9.8 to determine nuclear translocation (colocalization of GFP and Hoechst staining), Intensity (upper histogram), and the number of cells (lower histogram).


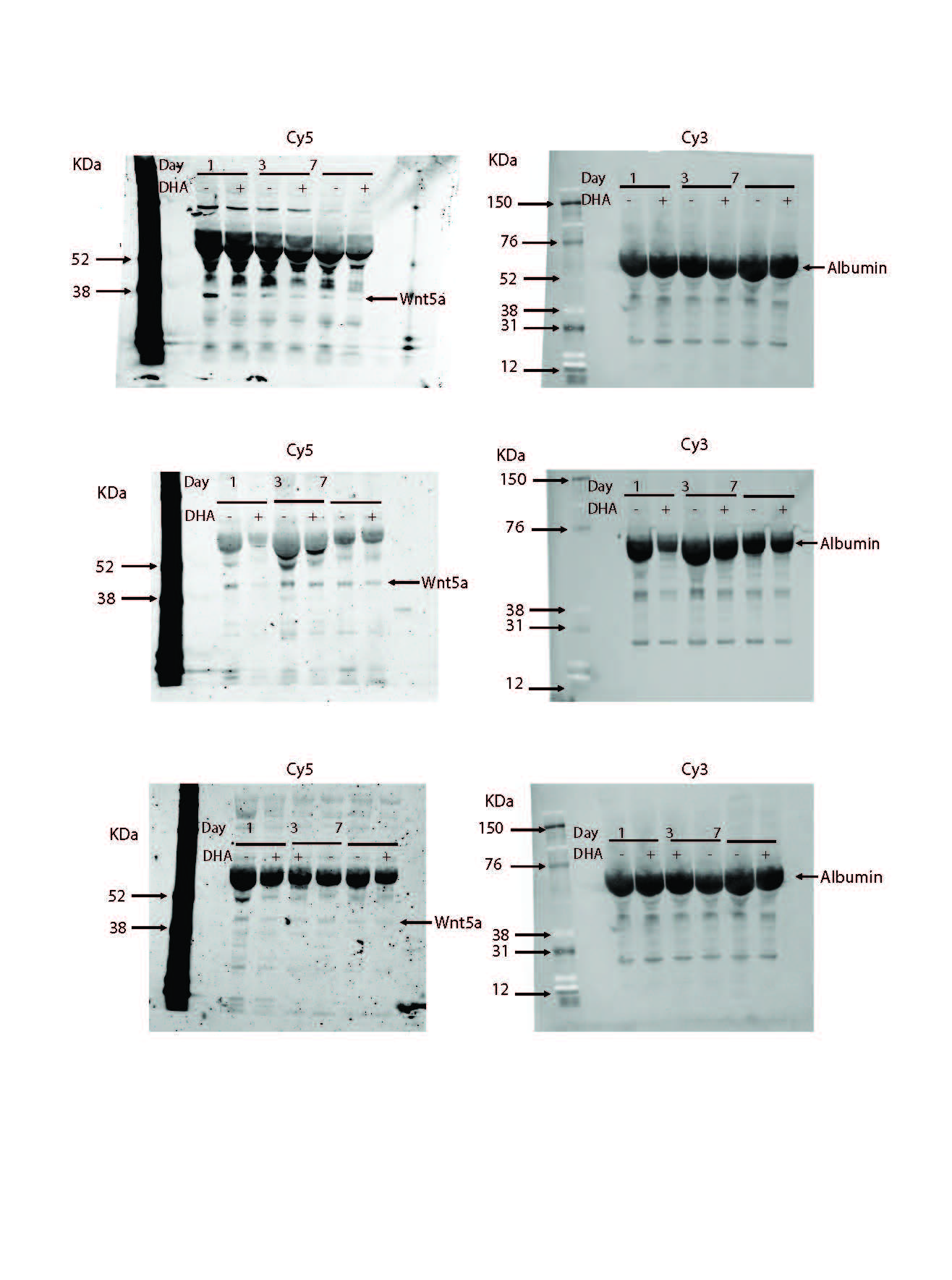


**Supplementary Fig. 12. Whole membranes for the western blots in Fig. 5g.** Wnt5a protein was quantified from Plasma samples obtained from MCAo rats treated and non-treated with DHA after 1, 3, and 7 days, using western blot. The standardization was performed using Albumin as housekeeping.


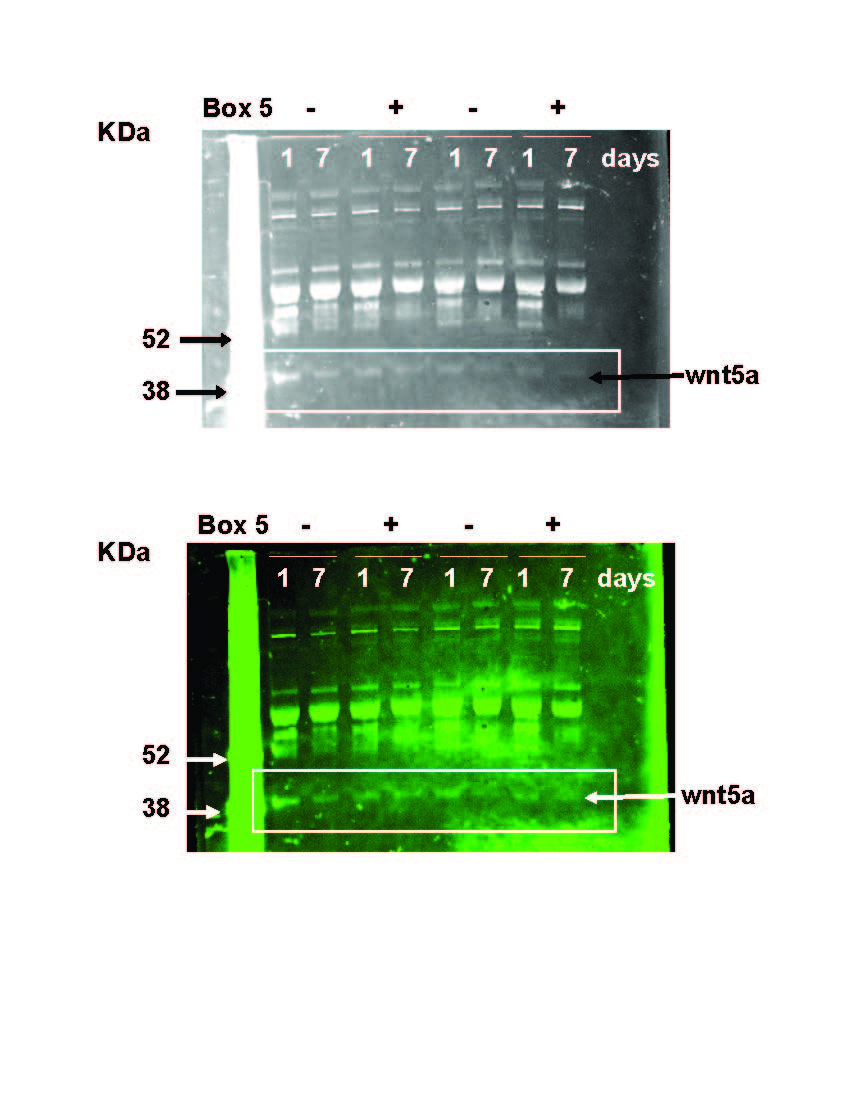


**Supplementary Fig. 13. Whole membrane for the western blots in Fig. 5h.** Wnt5a protein was quantified from Plasma samples obtained from MCAo rats treated and non-treated with DHA after 1 and 7 days, using western blot. The standardization was performed using Albumin as housekeeping.


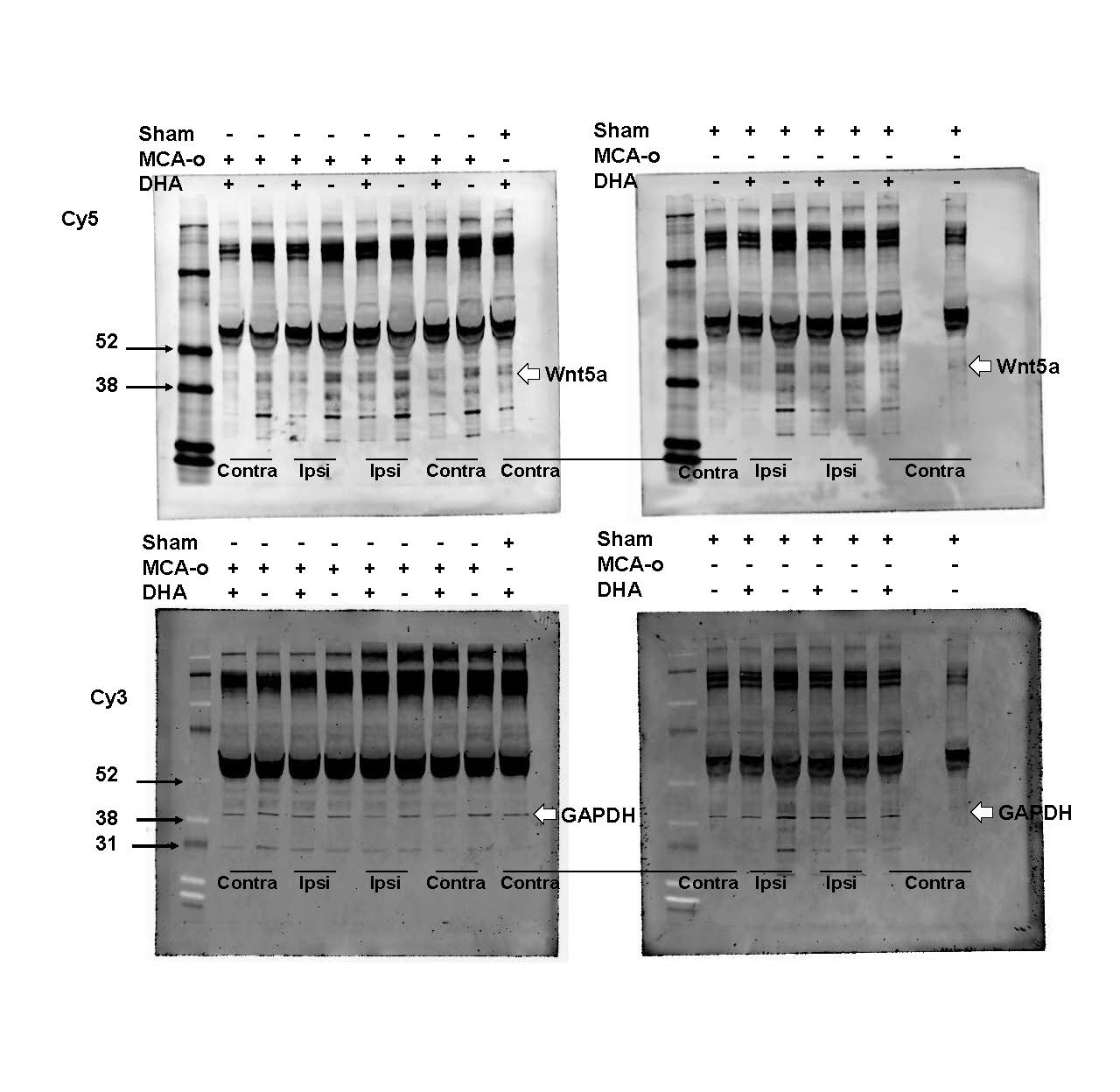


**Supplementary Fig. 14. Whole membranes for the western blots in Fig. 5i.** Wnt5a protein was quantified from cortical tissue samples obtained from MCAo and Sham rats treated and non-treated with DHA after 3 days using western blot. The standardization was performed using GAPDH as housekeeping.

**Supplementary Table 1.** TRED and TF bind analysis on the Promoter sequence (*2*), Related to **Fig. 4d-g**.

|  | Position / Sequence | TRED Score | TFBind Score |
| --- | --- | --- | --- |
| cREL | | | |
| Region 1 | **[192 .. 201] TAGAAATTCC** | 3.92 |  |
|  | **[214 .. 223] CCGGTTTTGC** | 2.21 |  |
|  | **[215 .. 224] CGGTTTTGCC** | 3.3 |  |
|  | 193 (+) SGGRNWTTCC TAGAAATTCC |  | 0.819522 |
|  | 194 (-) SGGRNWTTCC AGAAATTCCG |  | 0.844549 |
| Region 2 | **[357 .. 366] GGGACTTTGC** | 5.08 |  |
|  | 358 (+) SGGRNWTTCC GGGACTTTGC |  | 0.854004 |
| Region 3 | **[1445 .. 1454] GCGACTTTCA** | 4.12 |  |
| Region 4 | **[1550 .. 1559] CGGCATCTCC** | 3.3 |  |
|  | 1565 (-) SGGRNWTTCC GAAAAAGCCA | | 0.850945 |
| Region 5 | **[1945 .. 1954] CCTAATTACC** | 1.99 |  |
|  | 1939 (-) SGGRNWTTCC GGAAAGCCCT | | 0.887097 |
| Region 6 | **[2103 .. 2112] GGGCGCATCC** | 2.6 |  |
| Region 7 | **[2284 .. 2293] GGCGACTTCC** | 3.71 |  |
|  | 2285 (+) SGGRNWTTCC GGCGACTTCC | | 0.814516 |
| p65 | | | |
| Region 1 | **[192 .. 201] TAGAAATTCC** | 4.17 |  |
|  | 194 (-) GGGRATTTCC AGAAATTCCG |  | 0.868024 |
| Region 2 | **[357 .. 366] GGGACTTTGC** | 6.19 |  |
|  | 358 (+) GGGRATTTCC GGGACTTTGC |  | 0.861557 |
| Region 3 | **[1445 .. 1454] GCGACTTTCA** | 1.95 |  |
| Region 4 | **[1550 .. 1559] CGGCATCTCC** | 2.56 |  |
|  | 1551 (+) GGGRATTTCC CGGCATCTCC |  | 0.769102 |
|  | 1552 (-) GGGAMTTYCC GGCATCTCCC |  | 0.803246 |
| Region 5 | **[1937 .. 1946] TGGAAAGCCC** | 2.14 |  |
|  | 1938 (+) GGGRATTTCC TGGAAAGCCC |  | 0.782754 |
|  | 1939 (-) GGGRATTTCC GGAAAGCCCT |  | 0.86491 |
| Region 6 | **[2104 .. 2113] GGCGCATCCC** | 1.79 |  |
|  | 2105 (+) GGGRATTTCC GGCGCATCCC |  | 0.765749 |
| Region 7 | **[2284 .. 2293] GGCGACTTCC** | 2.72 |  |
|  | 2285 (+) GGGRATTTCC GGCGACTTCC |  | 0.771018 |
| NFkB/p50 | | | |
| Region 1 | 193 (-) NGGGACTTTCCA TAGAAATTCCGG |  | 0.760042 |
| Region 2 | **[357 .. 366] GGGACTTTGC** | 5.92 |  |
|  | 358 (+) GGGGATYCCC GGGACTTTGC |  | 0.750555 |
| Region 3 | **[1445 .. 1454] GCGACTTTCA** | 0.86 |  |
| Region 4 | **[1551 .. 1560] GGCATCTCCC** | 1.74 |  |
|  | 1551 (-) GGGGATYCCC CGGCATCTCC |  | 0.790315 |
|  | 1552 (-) GGGAMTTYCC GGCATCTCCC |  | 0.803246 |
| Region 5 | **[1937 .. 1946] TGGAAAGCCC** | 2.08 |  |
|  | 1939 (-) GGGGATYCCC GGAAAGCCCT |  | 0.79498 |
| Region 6 | **[2104 .. 2113] GGCGCATCCC** | 3.78 |  |
|  | 2105 (-) GGGGATYCCC GGCGCATCCC |  | 0.75522 |
| Region 7 | **[2285 .. 2294] GCGACTTCCT** | 2.95 |  |
|  | 2285 (+) GGGGATYCCC GGCGACTTCC |  | 0.754331 |

**Supplementary Table 2.** CpG islands detected by MethPrimer (*3*). Criteria: Island size > 100, GC Percent > 50.0, Obs/Exp > 0.6): 5 CpG island(s) were found in the sequence, Related to **Fig. 4e-g**.

|  | **Size** | **(Start - End)** |
| --- | --- | --- |
| Island 1 | 175 bp | (137 - 311) |
| Island 2 | 173 bp | (483 - 655) |
| Island 3 | 144 bp | (664 - 807) |
| Island 4 | 793 bp | (948 - 1740) |
| Island 5 | 338 bp | (1929 - 2266) |

**Supplementary Table 3.** DNA constructs and siRNAs, Related to **Fig. 2e, Fig. 3a, b, d, and i, Fig. 4b, c, Supplementary Fig. 4, Supplementary Fig. 5, Supplementary Fig. 6, Supplementary Fig. 7, Supplementary Fig. 9, Supplementary Fig. 10, Supplementary Fig. 11**.

| **Constructs** | **Type** | **Gene/protein** | **Reference/catalog number and company** |
| --- | --- | --- | --- |
| Wild Type cREL expression vector | REL (untagged)- UNIQUE VARIANT 1 of Human v-rel reticuloendotheliosis viral oncogene homolog (avian) (REL) | REL (NM_002908) | Origene True ORF Cat# SC126639 |
| NFkB reporter vector | 3 tandem copies of p65 binding sequence driving the expression of luciferase. |  | Qiagen, Cignal NFκB Reporter (luc) Kit: Cat # CCS-013L |
| Human Wnt5a  variant 1 | Open Reading frame |  | Origene True ORF Cat# SC126838 |
| Human FZD_5_ | Open Reading frame |  | Origene True ORF Cat# SC117952 |
| Human ROR2 | Open reading Frame |  | Origene True ORF Cat# SC117279 |
| TOP Flash | Super8XTOPflash construct M50, Beta-catenin reporter. TCF/LEF sites upstream of a luciferase reporter. | 7 TCF/LEF binding sites: AGATCAAAGGgggta, with TCF/LEF binding site in CAP letters, and a spacer in lower case, separating each copy of the TCF/LEF site). | Addgene repository Plasmid #12456  Zebrafish prickle, a modulator of noncanonical Wnt/Fz signaling, regulates gastrulation movements. Veeman et al, 2003) |
| FOP flash | M51 Super 8x FOPFlash (TOPFlash mutant) | 6 mutated TCF/LEF binding sites that were cloned into the pGL3 vector (Promega) | Addgene repository Plasmid #12457  (Veeman et al, 2003). |
| FZD_5_ siRNA | Human FZD_5_ 21-mer siRNA duplexes | Human FZD_5_ (NM_003468) | Silencer select Validated Ambion, Life Technologies –Thermo Cat# 4390824  ID: s15416 |
| FZD_1_ siRNA | Human FZD_1_ 21-mer siRNA duplexes | Human FZD_1_ (NM_003505) | Silencer select Validated Ambion, Life Technologies –Thermo Cat# 4390824  ID: s15835 |
| FZD_2_ siRNA | Human FZD_2_ 21-mer siRNA duplexes | Human FZD_2_ (NM_001466) | Silencer select Validated Ambion, Life Technologies –Thermo Cat# 4390824  ID: s5439 |
| FZD_4_ siRNA | Human FZD_4_ 21-mer siRNA duplexes | Human FZD_4_ (NM_012193) | Silencer select Validated Ambion, Life Technologies –Thermo Cat# 4390824  ID: s15840 |
| FZD_7_ siRNA | Human FZD_7_ 21-mer siRNA duplexes | Human FZD_7_ (NM_003507) | Silencer select Validated Ambion, Life Technologies –Thermo Cat# 4390824  ID: s15842 |
| FZD_8_ siRNA | Human FZD_8_ 21-mer siRNA duplexes | Human FZD_8_ (NM_031866) | Silencer select Validated Ambion, Life Technologies –Thermo Cat# 4392420  ID: s15845 |
| ROR2 siRNA | Human ROR2 21-mer siRNA duplexes | Human ROR2 (NM_004560) | Silencer select Ambion, Life Technologies–Thermo Cat# 4390824  ID: s9758 |
| Negative control siRNA Alexa Fluor 488 conjugated | Non-specific binding siRNA sequence | Proprietary | Allstars  Qiagen Cat#1027292 |

**Supplementary Table 4.** Primers information, Related to **Fig. 1c, e, and f, and Fig. 5f, j**.

| **Target** | **Sequence** | **Source** |
| --- | --- | --- |
| Rat Wnt5a | Forward primer  5’-TTACCCAAACCGGACTGTTA-3’ | RealTimePrimers.com |
|  | Reverse primer  5’-AGCCTTTTCGGTTCATCTCT-3’ |  |
| Human Wnt5a | Forward primer  5’-CAAAGCAACTCCTGGGCTTA-3’ | Campioni et al., 2008 (*4*) |
|  | Reverse primer  5’-CCTGCTCCTGACCGTCC-3’ |  |
| Rat Chemokine C-X-C motif ligand 1 (Cxcl1) NM_030845 | Forward primer  5’-GCGGAGAGATGAGAGTCTGG-3’ | RealTimePrimers.com |
|  | Reverse primer  5’-TCCAAGGGAAGCTTCAACAC-3’ |  |
| Rat ACTB NM_031144 | Forward primer  5’-CACACTGTGCCCATCTATGA-3’ | RealTimePrimers.com |
|  | Reverse primer  5’-CCGATAGTGATGACCTGACC-3’ |  |
| Rat TNFα | Forward primer  5’- ACCACGCTCTTCTGTCTACTG -3’ | Ohtomo et al., 2010 (*5*) |
|  | Reverse primer  5’- CTTGGTGGTTTGCTACGAC -3’ |  |
| Rat IL6 | Forward primer  5’-CTTCCTACCCCAACTTCCAA-3’ | RealTimePrimers.com |
|  | Reverse primer  5’-ACCACAGTGAGGAATGTCCA-3’ |  |
| Rat B2m | Forward primer  5’-TGCTACGTGTCTCAGTTCCA-3’ | RealTimePrimers.com |
|  | Reverse primer  5’-GCTCCTTCAGAGTGACGTGT-3’ |  |
| Rat MMP13 | Forward primer  5’-CCTCTTCTTCTCAGGGAACC-3’ | RealTimePrimers.com |
|  | Reverse primer  5’-GGAATTTGTTGGCATGACTC-3’ |  |
| Rat MMP9 | Forward primer  5’-ACTTCTGGCGTGTGAGTTTC-3’ | RealTimePrimers.com |
|  | Reverse primer  5’-TGTATCCGGCAAACTAGCTC-3’ |  |
| Rat MMP2 | Forward primer  5’-CTTCAGGTTCTCCAGCATGA-3’ | RealTimePrimers.com |
|  | Reverse primer  5’-CCGTAAGGGAGACACCAGAT-3’ |  |
| Rat IL-1β | Forward primer  5’- TCAGGAAGGCAGTGTCACTCATTG -3’ | Nakazawa et al., 2011 |
|  | Reverse primer  5’- ACACACTAGCAGGTCGTCATCATC -3’ |  |
| Rat ICAM1 | Forward primer  5’- CTGTCAAACGGGAGATGAATGGT -3’ | Ammirante et al., 2010 (*6*) |
|  | Reverse primer  5’- TCTGGCGGTAATAGGTGTAAATGG -3’ |  |
| Rat MCP1 | Forward primer  5’-ATGCAGGTCTCTGTCACGCTTCTG-3’ | Nakazawa et al., 2006 (*7*) |
|  | Reverse primer  5’- GACACCTGCTGCTGGTGATTCTCTT-3’ |  |
| Rat E-Selectin | Forward primer  5’-TGCGATGCTGCCTACTTGTG-3’ | Hannawa et al., 2005 (*8*) |
|  | Reverse primer  5’-AGAGAGTGCCACTACCAAGGGA-3’ |  |
| Rat Ywhaz | Forward primer  5’- GATGAAGCCATTGCTGAACTTG -3’ | Gubern et al., 2009 (*9*) |
|  | Reverse primer  5’- GTCTCCTTGGGTATCCGATGTC -3’ |  |
| Rat Sdha | Forward primer  5’- TCCTTCCCACTGTGCATTACAA -3’ | Gubern et al., 2009 (*9*) |
|  | Reverse primer  5’- CGTACAGACCAGGCACAATCTG -3’ |  |
| Human cREL | Forward primer  5'-CAGGAGGAAGAGCAGTCGTC-3' | Calandria et al., 2015 (*10*) |
|  | Reverse primer  5'- GCAGGAATCAATCCATTCAA-3' |  |
| Human Frizzled 1 receptor | Forward primer  5’-CAGCACAGCACTGACCAAAT-3’ | doi: [10.1371/journal.pone.0015526](https://doi.org/10.1371/journal.pone.0015526) |
|  | Reverse primer  5’-GTGAGCCGACCAAGGTGTAT-3’ |  |
| Human Frizzled 2 receptor | Forward primer  5’-CGATGGTTCCATGTTCTTCTC-3’ | doi: [10.1096/fj.201700144R](https://dx.doi.org/10.1096%2Ffj.201700144R) |
|  | Reverse primer  5’-GACCAGGTGAGGATCCAGAG-3’ |  |
| Human Frizzled 4 receptor | Forward primer  5’-TCTTCTCTGTGCACATTGGC-3’ | doi: [10.1186/s12876-019-0957-5](https://dx.doi.org/10.1186%2Fs12876-019-0957-5) |
|  | Reverse primer  5’-GACAACTTTCACACCGCTCA-3’ |  |
| Human Frizzled 7 receptor | Forward primer  5’-GCCAGCTTGTGCCTAATAGAA-3’ | doi: [10.1096/fj.201700144R](https://dx.doi.org/10.1096%2Ffj.201700144R) |
|  | Reverse primer  5’-AGCCGGGAGAAACTCACAG-3’ |  |
| Human Frizzled 8 receptor | Forward primer  5’-GTCCACCCTCCTCAGCCAAC-3’ | doi: [10.1038/nature22306](https://dx.doi.org/10.1038%2Fnature22306) |
|  | Reverse primer  5’-ACCCAGCCCCTTTTCCTCCATT-3’ |  |
| Human Frizzled 5 receptor | Forward primer  5’-AGCCCTTCGTGCCCATTCTG-3’ | Designed by Calandria JM using Primer Blast |
|  | Reverse primer  5’-CTCGTCGGCACTGAAGGACG-3’ |  |
| Human ROR2 | Forward primer  5’-CTGGCTCCAGAAAATTCAGA-3’ | Designed by Calandria JM using Primer Blast |
|  | Reverse primer  5’-CTCTCAGTGTCCCGGACTTC-3’ |  |

**Supplementary Table 5.** ChIP assay primers for SYBR green-based real-time PCR, Related to **Fig. 4e-g**.

| **Promoter** | **Primers** | **Sequence** | |
| --- | --- | --- | --- |
|  |  | **Forward** | **Reverse** |
| Wnt5a Promoter A | A1 | 5'-GCATCCCACTACCCAAGTCC-3' | 5'-GCTGCCTTGACATGGAACCTCA-3' |
|  | A2 | 5'-CAGCAATAAGTTCCGGGGCG-3' | 5'-GCTTTGGGGCCACAGAACAATC-3' |
|  | A3 | 5'-GCCTCTCCGTGGAACAGTTGC-3' | 5'-GATGCGCCCAGGAATGG-3' |
|  | A4 | 5'-CGCCAGTGCCCGCTTCAG-3' | 5'-CAGCCGAGGAATCCGAGC-3' |

References

1. J. M. Calandria, V. L. Marcheselli, P. K. Mukherjee, J. Uddin, J. W. Winkler, N. A. Petasis, N. G. Bazan, Selective Survival Rescue in 15-Lipoxygenase-1-deficient Retinal Pigment Epithelial Cells by the Novel Docosahexaenoic Acid-derived Mediator, Neuroprotectin D1. *J Biol Chem*. **284**, 17877–17882 (2009).

2. K. S. Katula, N. B. Joyner-Powell, C.-C. Hsu, A. Kuk, Differential regulation of the mouse and human Wnt5a alternative promoters A and B. *DNA Cell Biol.* **31**, 1585–1597 (2012).

3. L.-C. Li, R. Dahiya, MethPrimer: designing primers for methylation PCRs. *Bioinformatics*. **18**, 1427–1431 (2002).

4. M. Campioni, V. Ambrogi, E. Pompeo, G. Citro, M. Castelli, E. P. Spugnini, A. Gatti, P. Cardelli, L. Lorenzon, A. Baldi, T. C. Mineo, Identification of genes down-regulated during lung cancer progression: A cDNA array study. *J Exp Clin Cancer Res*. **27**, 38 (2008).

5. S. Ohtomo, Y. Izuhara, M. Nangaku, T. Dan, S. Ito, C. van Ypersele de Strihou, T. Miyata, Body weight control by a high-carbohydrate/low-fat diet slows the progression of diabetic kidney damage in an obese, hypertensive, type 2 diabetic rat model. *J Obes*. **2010** (2010), doi:10.1155/2010/136502.

6. M. Ammirante, J.-L. Luo, S. Grivennikov, S. Nedospasov, M. Karin, B-cell-derived lymphotoxin promotes castration-resistant prostate cancer. *Nature*. **464**, 302–305 (2010).

7. T. Nakazawa, C. Nakazawa, A. Matsubara, K. Noda, T. Hisatomi, H. She, N. Michaud, A. Hafezi-Moghadam, J. W. Miller, L. I. Benowitz, Tumor necrosis factor-alpha mediates oligodendrocyte death and delayed retinal ganglion cell loss in a mouse model of glaucoma. *J. Neurosci.* **26**, 12633–12641 (2006).

8. K. K. Hannawa, J. L. Eliason, D. T. Woodrum, C. G. Pearce, K. J. Roelofs, V. Grigoryants, M. J. Eagleton, P. K. Henke, T. W. Wakefield, D. D. Myers, J. C. Stanley, G. R. Upchurch, L-selectin-mediated neutrophil recruitment in experimental rodent aneurysm formation. *Circulation*. **112**, 241–247 (2005).

9. C. Gubern, O. Hurtado, R. Rodríguez, J. R. Morales, V. G. Romera, M. A. Moro, I. Lizasoain, J. Serena, J. Mallolas, Validation of housekeeping genes for quantitative real-time PCR in in-vivo and in-vitro models of cerebral ischaemia. *BMC Mol. Biol.* **10**, 57 (2009).

10. J. M. Calandria, A. Asatryan, V. Balaszczuk, E. J. Knott, B. K. Jun, P. K. Mukherjee, L. Belayev, N. G. Bazan, NPD1-mediated stereoselective regulation of BIRC3 expression through cREL is decisive for neural cell survival. *Cell Death Differ*. **22**, 1363–1377 (2015).
